# β-PruNet: An Explainable, PSO-Optimized Degree-Centrality-Pruned Regularized Extreme Learning Machine for Lightweight Household Appliance Recognition

**DOI:** 10.3390/biomimetics11100722

**Published:** 2026-10-10

**Authors:** Ömer Faruk Alçin, Muhammed Buğracan Özküçük, Muzaffer Aslan, İlhami Sel

**Affiliations:** 1 Department of Software Engineering, Faculty of Engineering, Inonu University, 44210 Malatya, Türkiye; 2 Department of Electrical and Electronics Engineering, Faculty of Engineering and Natural Sciences, Malatya Turgut Ozal University, 44210 Malatya, Türkiye; bugracan.ozkucuk@ozal.edu.tr; 3 Department of Electrical and Electronics Engineering, Faculty of Engineering and Architecture, Bingol University, 12100 Bingol, Türkiye; muzafferaslan@bingol.edu.tr; 4 Department of Computer Engineering, Faculty of Engineering, Inonu University, 44210 Malatya, Türkiye; ilhami.sel@inonu.edu.tr

**Keywords:** appliance recognition, ordinal patterns, regularized extreme learning machine, particle swarm optimization, synaptic pruning, explainable artificial intelligence

## Abstract

Accurate recognition of individual appliances from their own power consumption profiles underpins non-intrusive load monitoring (NILM), yet deep learning models often incur high computational overhead and lack operational interpretability. This study proposes a new explainable and lightweight model named β-PruNet to recognize household appliances using daily power consumption signals. The task addressed here is appliance classification from submetered profiles rather than disaggregation of an aggregate household signal, so the model is positioned as the recognition stage of a NILM pipeline. Each 24 h signal (86,400 samples) is transformed into a 720-bin ordinal pattern (OP) distribution, and the distribution is summarized by 27 statistical and spectral descriptors over 48 sliding windows. A compact class-weighted convolutional neural network extracts 128 embedding features. Drawing inspiration from collective swarm intelligence and biological synaptic pruning, a particle swarm optimization (PSO)-tuned regularized extreme learning machine (R-ELM) classifier with beta-weight centrality pruning conducts classification via a diverse softmax ensemble. On an imbalanced 18-class benchmark containing 916 daily appliance profiles, β-PruNet achieved 98.9% classification accuracy and a 97.6% macro F1-score while pruning 87.3% of hidden neurons (from 1500 to 191) without accuracy loss. Post hoc explainability analyses using SHAP, LIME, and Grad-CAM confirmed that the pruning criterion strongly correlates with Shapley feature attribution (r = 0.974). These findings indicate that integrating symbolic dynamics with bio-inspired pruning delivers a computationally efficient, highly accurate, and transparent solution suitable for edge-oriented energy management systems. The theoretical contribution is that the magnitude of a hidden unit’s learned output weights provides a usable ranking signal at no additional computational cost beyond the closed-form solution already obtained. The practical contribution is that this signal allows the classifier head to be compressed by 87.3% without a measurable loss in accuracy.

## 1. Introduction

Electrical energy consumption across residential buildings has grown steadily, establishing appliance-level load monitoring as a fundamental requirement for demand-side management, consumer energy feedback, and smart grid operation [1,2,3]. Since installing a separate meter for every device is costly and impractical, non-intrusive load monitoring (NILM) and appliance recognition from power consumption signals have become popular research areas [4,5]. However, deep learning models introduced to overcome the limited accuracy of traditional techniques often entail substantial computational overhead and suffer from a “black-box” nature that obscures the interpretability of their decisions. To overcome these limitations, this study proposes an explainable, lightweight, and bio-inspired appliance recognition model termed β-PruNet. The developed β-PruNet transforms each daily power signal into ordinal pattern (OP) distributions and classifies the resulting features through a regularized extreme learning machine (R-ELM) network governed by biomimetic principles. Specifically, it couples particle swarm optimization (PSO), drawing on the collective foraging and flocking dynamics of swarm intelligence, to explore the hyperparameter search space, with a beta-weight centrality pruning mechanism inspired by biological synaptic pruning to discard non-essential hidden units, thereby achieving high classification fidelity under minimal computational complexity. The task is appliance classification from submetered, individual power profiles rather than disaggregation of an aggregate household signal, so the model is positioned as the recognition stage of a NILM pipeline rather than a complete NILM system.

The design of β-PruNet is biomimetic in a specific and load-bearing sense. The two mechanisms that keep the model accurate while making its classifier head small are modelled on biological strategies rather than invoked as decorative analogies. The first is developmental synaptic pruning. A mammalian cortex does not grow the connectivity it will ultimately use. It over-produces synapses during early development and then eliminates the weakly contributing ones, retaining roughly half of the peak count while preserving, and often improving, functional performance. The retained synapses are those carrying the largest and most reliable signal to downstream targets. β-PruNet reproduces this two-phase schedule directly. The randomized hidden layer is deliberately over-provisioned to 1500 units, an output-weight solution is obtained in closed form, and each hidden unit is then scored by the total magnitude of the weights it projects onto the output layer, which is its weighted degree in the hidden-to-output bipartite graph. Units with low projected weight are eliminated, and the remaining weights are re-solved, which parallels the way biological elimination is followed by strengthening of the surviving connections. The parallel is the source of the design and is not a claim of mechanistic equivalence. No biological quantity is measured here, and the comparison is offered as an inspiration in the sense used in the biomimetics literature. The engineering outcome is that 87.3% of the units of the classifier head are removed with zero accuracy degradation, because what is removed carried little signal in the first place.

The second mechanism is collective search. Particle swarm optimization abstracts the foraging behaviour of flocks and schools, in which each individual balances its own best experience against the best position known to the group, and the colony converges on good resources without any individual surveying the whole space. In β-PruNet, this governs the choice of hidden-layer size, pruning ratio, regularization strength, weight scale, and activation, so the configuration of the head is discovered by a swarm rather than fixed by hand. The head is a regularized extreme learning machine (R-ELM), with no direct input-to-output shortcut. A third, lighter correspondence runs through the front end. The ordinal pattern transform discards absolute amplitude and encodes only the rank order of successive samples, which is the same economy that rank-order coding exploits in early sensory pathways, where the sequence in which neurons fire carries the stimulus identity and the absolute rates are largely discarded. Taken together, the pipeline maps stage by stage onto a biological template, moving through rank-order sensory encoding, over-provisioned connectivity, activity-dependent elimination, and swarm-level search over the resulting design. This is the sense in which the present work belongs to biomimetics. The compression and the search are not engineering conveniences bolted onto a deep network; they are the biological principles doing the computational work.

### 1.1. Literature Review

Before data-driven classifiers became standard, appliance recognition rested on event-based and rule-based analysis of the power signal. The original formulation detected step changes in real and reactive power and matched each transition against a catalogue of steady-state (P, Q) signatures, assigning events to appliances by nearest-neighbour or combinatorial search. Later refinements added harmonic content, transient shapes and voltage-current trajectories to the signature set, and replaced exhaustive matching with hidden Markov models, clustering, or hand-tuned decision thresholds. These methods are transparent and cheap, and they remain competitive whenever the appliance set is small, and the signatures are well separated. Their limitations are equally well established. Steady-state (P, Q) signatures overlap heavily for devices of similar rating, so resistive loads of comparable power are routinely confused. The thresholds that govern event detection must be retuned for every installation and degrade under noise and partial loading. Multi-state and continuously variable appliances violate the on/off event model outright. Combinatorial matching also scales poorly as the number of candidate appliances grows.

Appliance recognition studies generally use public benchmark datasets such as PLAID [6], WHITED [7], and UK-DALE [8], alongside toolkits such as NILMTK [9] and plug-load measurement collections [10]. When examining deep learning methodologies evaluated on these benchmarks, residual convolutional neural networks (Res-CNNs) processing two-dimensional load signatures have achieved classification accuracies of 97.43%, 95.99%, and 98.14% on PLAID, WHITED, and HRAD, respectively [11]. Moreover, sequence-to-sequence and attention-based architectures have been widely applied to load disaggregation and appliance identification problems [12,13,14,15,16,17]. Recent efforts demonstrate a clear shift toward hybrid frameworks; specifically, combinations of Transformers with temporal convolutions [18], CNN–BilSTM–Transformer pipelines [19], attention-guided CNN-BiLSTM networks [20], and energy-conserving LSTM designs [21,22] have been reported to outperform their single-family baselines.

Input representation is equally decisive for recognition accuracy. Transforming raw time-series inputs into domain-specific representations such as FFT-based spectral features [23], discrete wavelet multi-task representations [24], Gramian angular field images [25], current harmonic distortion images [26], conventional or color-coded voltage-current (V–I) trajectories [27,28], fractal descriptors for low-power devices [29], and advanced engineered feature sets [30,31] have markedly enhanced diagnostic discriminability. On the classical side, shallow classifiers such as k-nearest neighbor (k-NN) remain competitive when coupled with well-crafted feature descriptors [32,33,34]. For the daily household appliance profile dataset used in this study, the highest benchmarks have been established by hybrid workflows: coupling LSTM-derived representations with k-NN, ensemble bagged trees, and support vector machines yielded accuracies of 98.25%, 97.81%, and 97.38%, respectively [35], while an STFT-driven local phase quantization hybrid framework attained 98.69% [36]. However, evaluation practices in the literature remain heterogeneous, and reported accuracies must be interpreted carefully in light of the validation protocol, sampling rate, and model generalization [4,37,38,39,40,41].

The works cited above also trace a visible progression rather than a flat catalogue. Early methods mapped the power signal onto an image, such as a Gramian angular field or a voltage-current trajectory, and classified the image with a convolutional network. This made the representation geometric but left the temporal extent of one image fixed at a single event. Sequence-based methods followed, replacing the single image with sequence-to-sequence or sequence-to-point disaggregation, which extended the representation across the whole observation window at the cost of a larger model. The most recent studies move in the opposite direction, pairing a lightweight or randomized classifier with a compact engineered or learned representation to recover efficiency without losing the gains of the sequence-based stage. This progression, from image-based classification to sequence disaggregation to lightweight hybrid pipelines, is the context in which the present study is positioned, and it reflects a broader shift in interest toward recognition systems that remain accurate while becoming cheaper to train and to deploy.

### 1.2. Literature Gaps

The key limitations identified in the existing literature are listed below:

• Ordinal pattern and other symbolic complexity-based representations have not been reported for daily appliance profile classification, although information-theoretic analyses indicate that load signals carry rich structural information [42,43].
• The best published results on the used dataset family are hybrid feature-plus-classical-classifier pipelines [35,36]; however, these models do not provide model compression or explainability analyses.
• Pruning criteria of randomized networks are generally justified only by accuracy, and no study has validated the pruning decision itself with Shapley-based attribution.
• Class imbalance handling is underreported in appliance recognition studies, although sample sizes per appliance are highly uneven [44,45].

### 1.3. Motivation and Proposed Model

The primary objective of this study is to develop an accurate, lightweight, and explainable framework for household appliance recognition. End-to-end deep learning architectures typically incur high computational demands and rely on massive datasets, while their black-box nature restricts the interpretability and auditing of decisions. To overcome these challenges, a hybrid approach is adopted. In the proposed β-PruNet, the signal-level workload is alleviated via a noise-robust symbolic transformation: each 24 h power signal is mapped into an order-5 ordinal pattern (OP) distribution [43,46] using the efficient algorithm of Unakafova and Keller [47]. A compact class-weighted CNN is utilized strictly as a one-time feature extractor, and the final decision is carried out by a closed-form, ridge-solved R-ELM network [48,49,50]. The hidden layer is pruned via biologically inspired beta-weight centrality and tuned through PSO [51]. Consequently, the deployed classifier retains only 191 hidden neurons and operates through a single matrix multiplication during inference.

The main novelties and contributions of this study are summarized as follows:

• To the best of our knowledge, this work is the first to introduce the full ordinal pattern distribution (720 bins) as a learnable input representation for daily appliance power profiles.
• A windowed distribution-map feature extraction strategy is introduced, treating the OP distribution as a pseudo-signal and extracting 27 statistical, spectral, and time-frequency descriptors across 48 sliding windows.
• A PSO-optimized beta-weight centrality pruning mechanism inspired by biological synaptic pruning is developed for R-ELM networks, and the pruning decision is validated post hoc through Shapley additive explanations (r = 0.974).
• The proposed β-PruNet achieved 98.9% classification accuracy and a 97.6% macro F1-score on a highly imbalanced 18-class benchmark, outperforming existing hybrid models reported on this dataset family.
• An 87.3% model compression (reducing hidden units from 1500 to 191) was accomplished with zero accuracy degradation, ensuring feasibility for resource-constrained edge deployment.
• A multi-level explainability framework (integrating SHAP, LIME, and Grad-CAM) is provided to systematically interpret the bio-inspired optimization, structural pruning, feature attribution, and signal-space dynamics.

## 2. Materials and Methods

**Figure 1:**
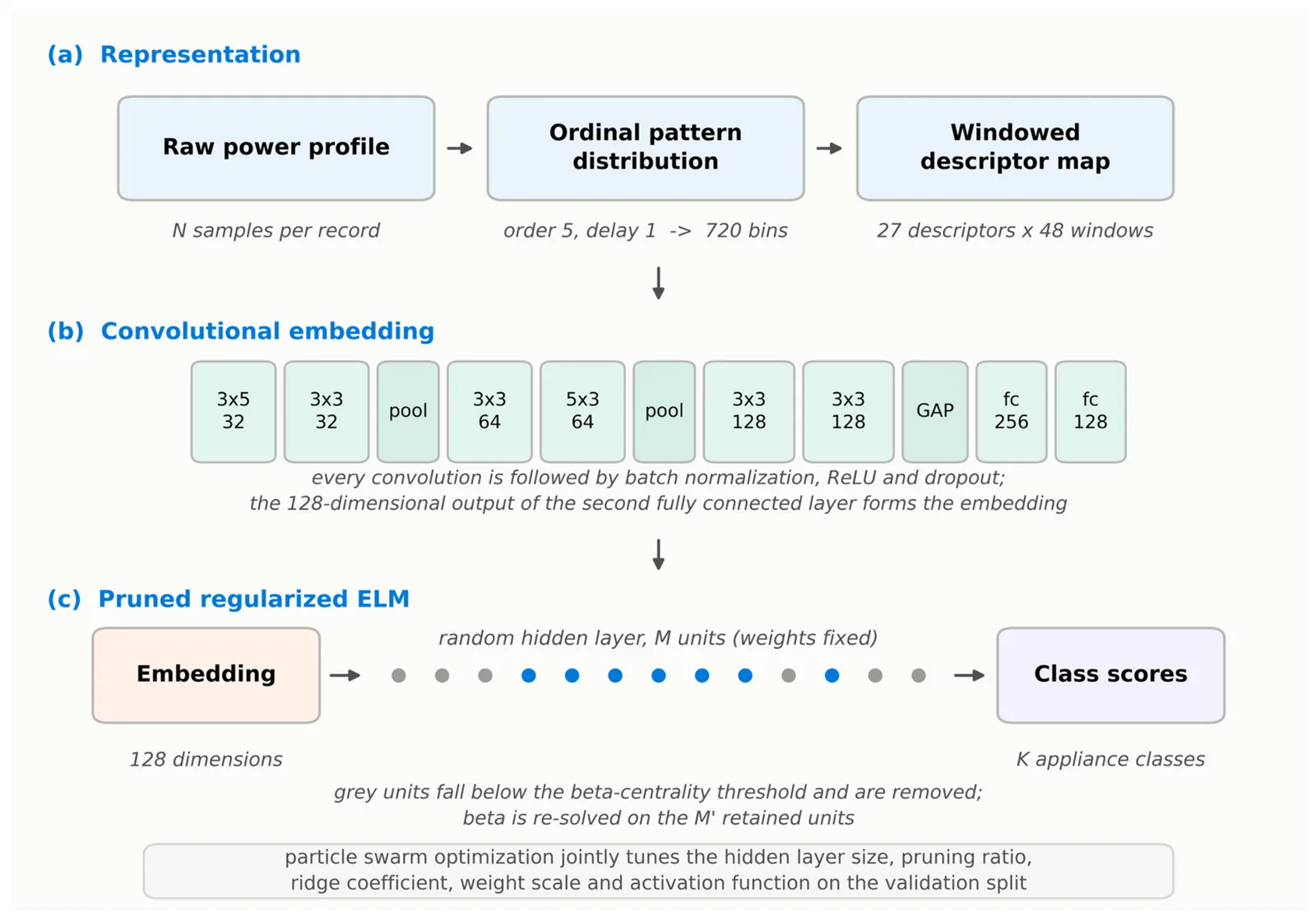
Flowchart of the study.

Figure 1 summarises the stages of the study and the order in which they are applied. The three bands trace the pipeline end to end. The representation chain converts the raw profile into a descriptor map, the convolutional network reduces that map to a 128-dimensional embedding, and the pruned classifier head reaches a decision using only the units retained after pruning. The architecture itself is summarised in panel (b) of the Figure 1. Six convolutional blocks (kernel sizes 3 × 5, 3 × 3, 3 × 3, 5 × 3, 3 × 3 and 3 × 3, with 32, 32, 64, 64, 128 and 128 channels, respectively) are interleaved with two pooling stages, each convolution followed by batch normalisation, ReLU and dropout, and the stack ends in global average pooling and two fully connected layers of 256 and 128 units. Panel (c) shows the classifier head, a randomized hidden layer connected to the embedding, scored by the beta-weight centrality criterion, with the retained units solved in closed form and the removed units excluded from the final model.

**Figure 2:**
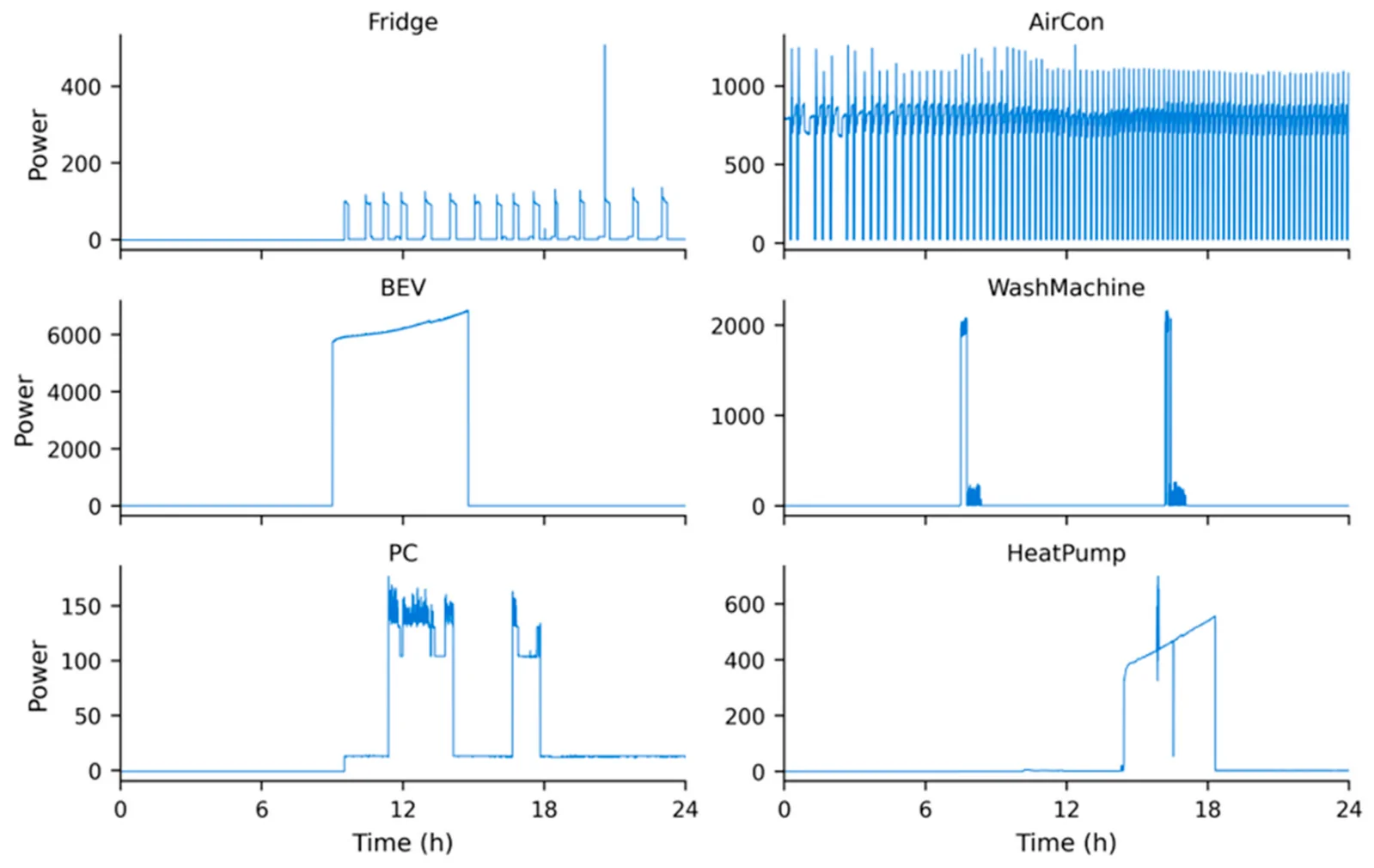
Representative 24 h power profiles of six appliance classes.

The dataset used in this work contains 916 appliance-level daily power consumption profiles collected from household devices [52]. Each profile is sampled at 1 Hz for 24 h; therefore, each observation contains 86,400 samples. The dataset covers 18 appliance classes and is strongly imbalanced: the largest class (Fridge) contains 168 profiles, while the smallest classes (Dryer and Always_on) contain only 6 profiles each. The class distribution of the dataset is given in Table 1, and representative daily profiles are shown in Figure 2.

A stratified hold-out protocol was applied: 20% of the observations (183 profiles) were reserved as the test set, and 10% of the remaining observations (73 profiles) were used as the validation set. The test set was used only once, for final reporting. Augmentation, the hyperparameter search, and the pruning-ratio selection are all performed using the training and validation splits only. An earlier version of the pruning-ratio sensitivity sweep selected over ratios using test metrics. That was an error, identified during this revision, and it was corrected so that the test split is touched exactly once, for the final reported evaluation.

A single partition fixes one particular assignment of profiles to the test set, so the hold-out result is reported alongside two resampling protocols that do not depend on that assignment. In the first, stratified five-fold cross-validation, the profiles are divided into five class-proportional folds. Each fold serves once as the test set while the remaining four supply the training and validation data, so the five test sets are disjoint and every profile is held out exactly once. In the second, the stratified hold-out split is repeated 30 times under independent seeds. The convolutional embedding, the hyperparameter search, the pruning step, and the closed-form solution are all repeated inside every fold and every repetition. The embedding in particular is retrained from scratch on the training part of each partition and is never shared between partitions, so no representation is fitted on profiles that later serve as test data, and no quantity reported in this work is selected on test data. The repeated-split and fold-level results are presented in Section 4.2. 

Household PQ is the primary dataset of this study. Every design decision, every hyperparameter range, and every component of the pipeline is fixed on it. A second, independent collection, PLAID 2018, is used as a supplementary evaluation dataset. Its purpose is to check whether the behaviour observed on the primary dataset is specific to that collection. The PLAID 2018 submetered collection is used exactly as distributed. It contains 1876 records of 16 appliance types sampled at 30 kHz, with a record length between 0.70 s and 20 s. Every record carries a location identifier, and the subset spans 56 distinct locations. The appliance labels are taken exactly as distributed, with no merging or relabelling. In the distributed metadata file metadata_submetered.json, Hairdryer has 246 records, Compact Fluorescent Lamp has 230, Fan has 220, Laptop has 216, Air Conditioner has 204, Microwave has 200, Incandescent Light Bulb has 157, Fridge has 108, Heater has 85, Vacuum has 83, Washing Machine has 75, Soldering Iron has 20, Coffee maker has 10, Water kettle has 10, Hair Iron has 10, and Blender has 2. We applied no filtering beyond this and did not use the aggregated portion of the collection. The ordinal pattern distribution has a fixed dimensionality regardless of record length, so no truncation or padding is required for the shorter PLAID records. The results obtained on this supplementary dataset are reported alongside the primary results later in this manuscript.

The six profiles illustrate markedly different consumption shapes, ranging from steady duty-cycled loads to brief high-amplitude pulses, the same qualitative contrast that the ordinal pattern distributions discussed in Section 3.1 are later shown to separate.

## 3. The Proposed β-PruNet

**Figure 3:**
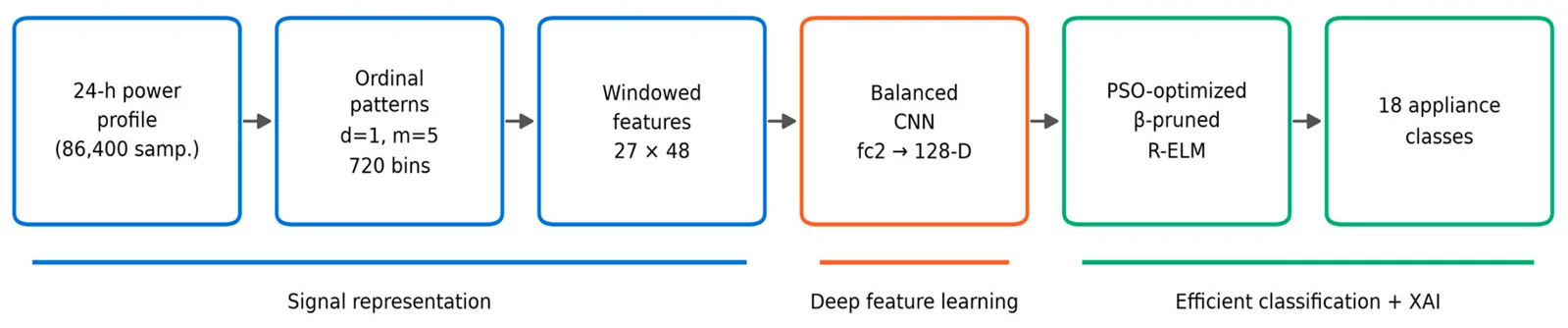
Graphical outline of the proposed β-PruNet.

The proposed β-PruNet is a four-phase model designed to achieve high recognition accuracy with minimal computational footprint, and the graphical outline of the model is shown in Figure 3. The pipeline bridges symbolic signal representation with bio-inspired randomized learning to ensure edge-level deployability. Specifically, the phases of the proposed β-PruNet are (i) ordinal pattern distribution extraction, (ii) windowed distribution-map feature generation, (iii) CNN-based feature embedding, and (iv) PSO-optimized pruned R-ELM classification with diverse ensemble voting. By replacing dense iterative backpropagation with symbolic mapping, structural pruning, and closed-form ridge solutions, the framework maintains rigorous interpretability across all inference stages.

The detailed operational steps of the proposed model are given below:

• Step 1: Read the daily power signal with 86,400 samples.
• Step 2: Compute the order-5, delay-1 ordinal pattern of each window of six consecutive samples using inversion-number encoding; obtain the normalized 720-bin OP distribution of the signal.
• Step 3: Apply median centering, linear detrending, and median absolute deviation scaling to the OP distribution.
• Step 4: Divide the 720-bin distribution into 48 overlapping windows (length 62, hop 14) and extract 27 descriptors from each window; obtain the 27 × 48 feature map.
• Step 5: Balance the training set by replication and augmentation (amplitude scaling, Gaussian noise, and circular shifting) and train the compact class-weighted CNN with the Adam optimizer [53].
• Step 6: Extract the 128-dimensional penultimate-layer (fc2) features of all observations.
• Step 7: Optimize the R-ELM hyperparameters (hidden size, prune ratio, ridge coefficient, weight scale, and activation) with PSO. Prune the hidden neurons with the lowest beta-weight centrality and re-solve the output weights.
• Step 8: Generate the final prediction with a 20-member diverse softmax ensemble, and produce the explainability reports (SHAP, LIME, and Grad-CAM).

### 3.1. Ordinal Pattern Distribution

**Figure 4:**
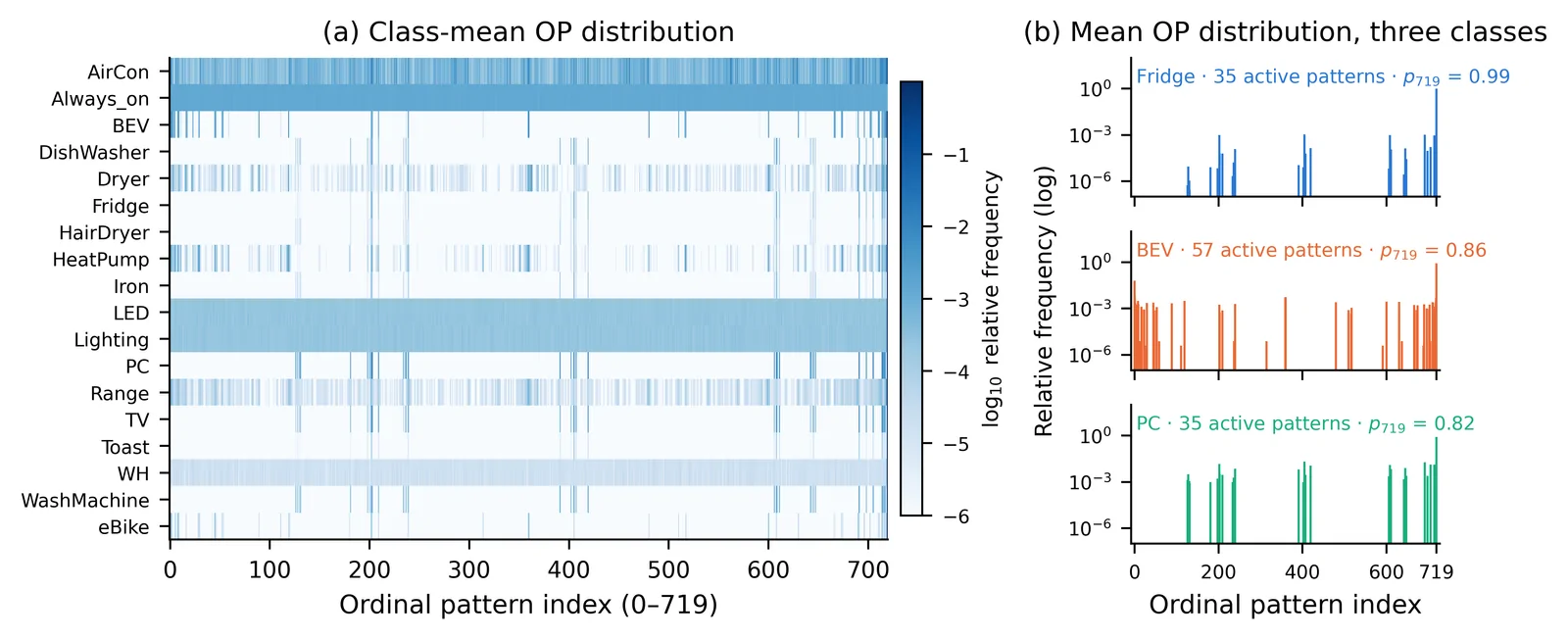
Class-mean ordinal pattern distributions: (**a**) heatmap over the 18 classes; (**b**) mean distributions of three classes on a logarithmic scale.

Ordinal patterns model the relative rank order of consecutive observations rather than their absolute amplitude values; consequently, they exhibit invariance to monotonic amplitude shifts and provide high robustness against measurement noise [43,54]. When selecting an embedding order *m* = 5 and time delay *τ* = 1, each sub-sequence of six consecutive samples (*m* + 1) is mapped to one of (*m* + 1)!=720 possible permutation states using the inversion-number algorithm proposed by Keller and Sinn [46]. This symbolic transformation is computed via the linear-time O(*N*) algorithm of Unakafova and Keller [47], thereby minimizing computational overhead. Normalizing the total of 86,395 local ranking states obtained across the sliding windows over a 24 h power signal (*N* = 86,400 samples) yields a 720-bin probability density histogram per profile. As illustrated in Figure 4, this representation yields distinct and discriminative symbolic signatures across periodic duty-cycled loads (Fridge, AirCon), sustained constant-consumption loads (BEV), and brief, bursty resistive loads (Toas, HairDryer).

### 3.2. Windowed Distribution-Map Feature Generation

**Figure 5:**
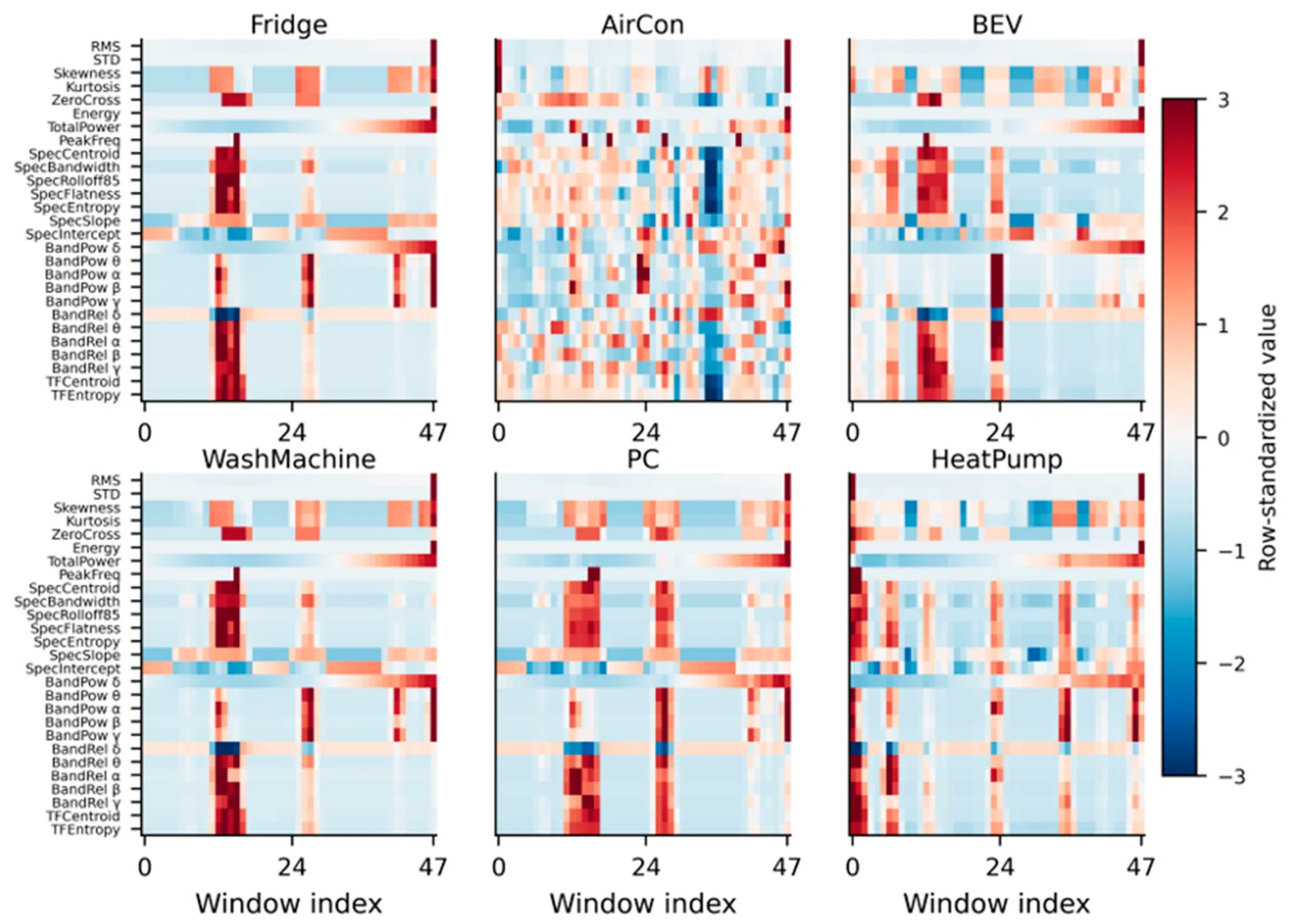
Examples of the 27 × 48 windowed distribution-map features.

The OP distribution is treated as a pseudo-signal of length 720. After median centering, linear detrending, and median absolute deviation scaling, the distribution is divided into 48 windows of length 62 with a hop of 14 samples. From each window, 27 descriptors are extracted: six time-domain statistics (root mean square, standard deviation, skewness, kurtosis, zero crossings, and energy), total periodogram power, eight spectral shape descriptors (peak frequency, centroid, bandwidth, 85% roll-off, flatness, entropy, log–log slope, and intercept), five absolute and five relative band powers, and two short-time Fourier-transform summaries. Hence, Figure 5 shows the resulting interpretable 27 × 48 feature map obtained for each observation, and the named rows of this map enable the Grad-CAM and permutation-importance analyses in Section 5.

### 3.3. CNN-Based Feature Embedding

A compact CNN with six convolutional layers (32-32-64-64-128-128 filters), batch normalization, two max-pooling layers, dropout, global average pooling, and two fully connected layers (256 and 128 neurons) was trained with a class-weighted cross-entropy loss. Before training, the training set was balanced to the majority class count: half of each deficit was filled by random replication and the other half by augmentation (±3% amplitude scaling, additive Gaussian noise with a standard deviation of 0.01, and ±2-window circular shifting), following the general oversampling principle of Chawla et al. [55]. The network was trained with the Adam optimizer (initial learning rate 10^−3^, batch size of 128, at most 120 epochs, and validation-loss patience). The 128-dimensional linear output of the penultimate layer was used as the observation embedding for the classification phase. Replication and augmentation serve complementary purposes. Replication corrects the class prior without adding new information, whereas augmentation adds variation but can drift away from the class manifold when very few genuine profiles are available. The two are therefore combined rather than used alone, so that the balancing procedure both restores the class prior and introduces genuine variation.

### 3.4. PSO-Optimized Beta-Centrality Pruned R-ELM

For the classification stage, we employ a randomized single-hidden-layer network trained in closed form. Given the input matrix *X* ∈ ℝ*^N^*^×^*^d^*, where *N* denotes the number of samples and *d* = 128 represents the feature embedding dimension, the hidden layer activation matrix *H* ∈ ℝ*^N^*^×^*^L^* is formulated as:

(1) *H* = *ϕ*(*XW* + *b*)

where *W* ∈ ℝ*^d^*^×^*^L^* is the input weight matrix randomly generated from a continuous uniform distribution, *b* ∈ ℝ^1×^*^L^* is the randomly assigned bias vector (broadcast across all *N* rows), *L* denotes the initial number of hidden neurons (*L* = 1600), and *ϕ*(⋅) represents the element-wise nonlinear activation function (such as radial basis function, sine, or sigmoid, as explored during hyperparameter optimization). Unlike standard backpropagation networks, *W* and *b* remain fixed throughout the training process.

The design matrix used throughout this work is *G* = *H* ∈ ℝ*^N^*^×^*^L^*. No direct input-to-output shortcut is used, so the classifier is a regularized extreme learning machine (R-ELM) rather than a random vector functional link network. The output weight matrix *β* is then obtained analytically via ridge regression [48,49]:

(2) $$\beta ={\left(G^{T}G+\mathrm{\lambda I}\right)}^{-1}G^{T}T$$

where *λ* > 0 is the regularization parameter, *I* is the identity matrix, and *T* ∈ {0,1}*^N^*^×^*^C^* denotes the one-hot target matrix across *C* = 18 appliance categories. To prune redundant hidden units, the beta-weight centrality of each hidden neuron *j* ∈ {1, …, *L*} is defined as the ℓ_1_-norm of its outgoing weights, ‖*β_j_*_,:_‖ _1_. Hidden neurons exhibiting the lowest centrality values are discarded, and β is recomputed using only the surviving columns of *G*.

**Figure 6:**
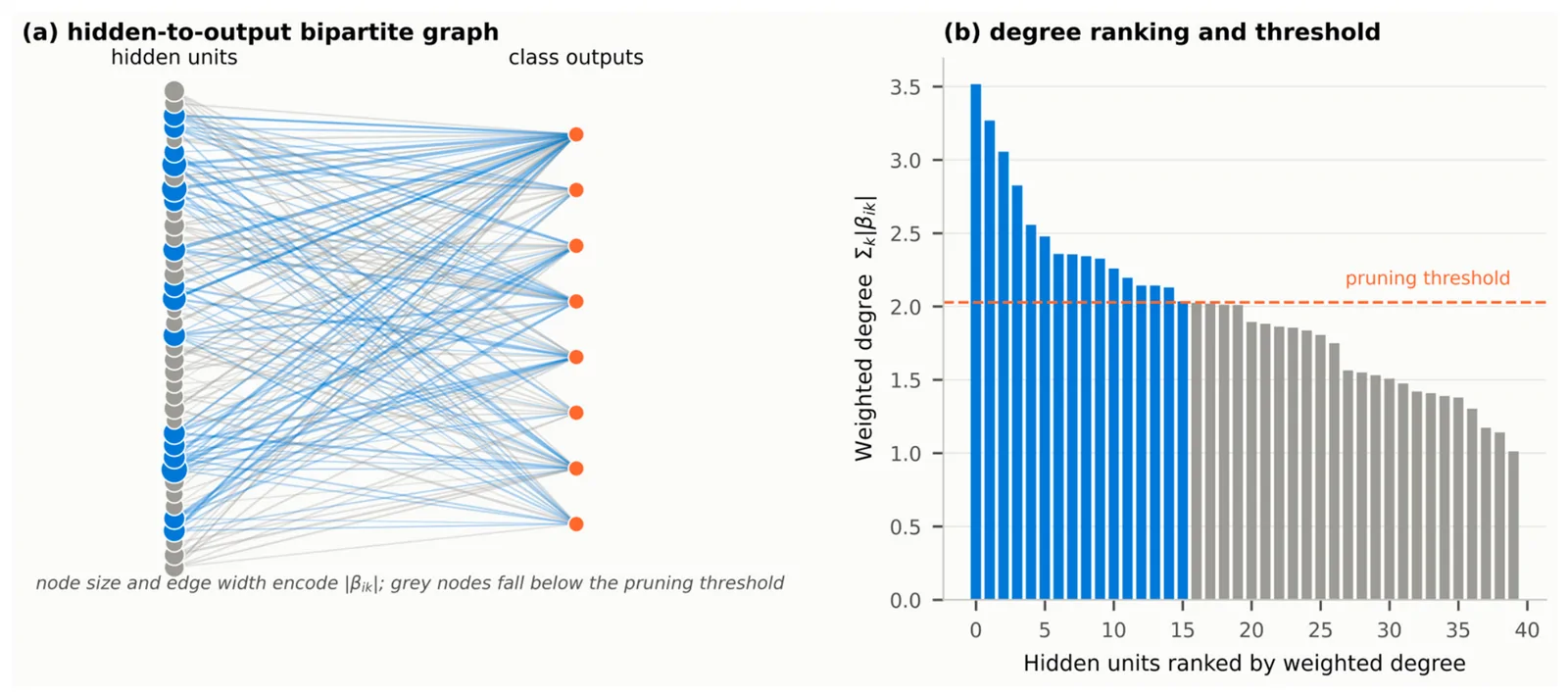
The pruning criterion as a weighted degree on the hidden-to-output bipartite graph. (**a**) Blue and grey nodes represent retained and pruned hidden neurons, respectively; orange nodes represent output classes, while connecting lines represent weighted connections. (**b**) Hidden neurons ranked by weighted degree, with the dashed orange line indicating the pruning threshold.

Figure 6 makes explicit the bipartite graph on which the pruning criterion is defined. Figure 6a shows the hidden-to-output graph, where node size and edge width encode the magnitude of the learned output weight between a hidden unit and a class output. Figure 6b shows the hidden units ranked by their weighted degree, together with the pruning threshold.

The key hyperparameters, including the initial hidden layer size, pruning ratio, *λ*, weight scale factor, and activation function—are tuned via Particle Swarm Optimization (PSO) configured with 18 particles, an inertia weight of 0.72, and acceleration constants *c*_1_ = *c*_2_ = 1.49. The optimization fitness objective jointly considers validation accuracy, macro F1-score, and the model compression ratio. Following global search, a narrow local grid search refines the optimal configuration. The final classification is produced by an ensemble of 20 perturbed models constructed from the top five validation setups. As Table 2 shows, the network shrinks from 1500 to 191 hidden neurons without sacrificing classification performance.

Having specified these hyperparameters, it is worth situating the pruning criterion itself within the broader literature. Pruning randomized networks is not new, and it is worth being precise about what is and is not novel here. The Optimally Pruned Extreme Learning Machine (OP-ELM) [56] ranks hidden units by multiresponse sparse regression and truncates using a leave-one-out error estimate, and the Tikhonov-regularized OP-ELM (TROP-ELM) [57] adds a second regularization stage to the same procedure. The Pruned Extreme Learning Machine (P-ELM) [58] and related variants apply statistical significance tests to the hidden responses, while constructive and destructive ELM variants add or remove units while monitoring validation error. In the wider network-compression literature, magnitude pruning removes the smallest input weights, activation-based pruning removes the least-activated units, and optimal brain damage (OBD) [59] and optimal brain surgeon (OBS) [60] use the Hessian of the training loss to estimate the cost of removing each unit.

The criterion used here, the weighted degree of a hidden unit in the trained output layer, is related to this literature but differs from it in cost rather than in kind. Because the output layer of R-ELM is linear and solved in closed form, the saliency that OBD and OBS approximate with the Hessian is available here exactly, as the magnitude of the already-computed output weights, so the comparison with these criteria is made on equal terms rather than as an approximation. No new graph-theoretic construction is claimed. The criterion is the degree of a hidden unit in the bipartite graph that the trained output layer already defines between hidden units and class outputs.

Reproducibility of this search matters as much as the criterion itself, so it is specified in full here. The search varies five decision variables. The initial hidden units range from 100 up to the smaller of 1500 and 90% of Ntrain, where Ntrain is the number of training rows after the balancing procedure of Section 3.3. On the primary dataset, Ntrain is 2178, which is 121 rows for each of the 18 classes, so 90% of Ntrain is 1960 and the cap of 1500 is the binding one. These 2178 rows come from 660 original training profiles through replication and augmentation, and they are not 2178 independent observations. The pruning ratio ranges from 0.20 to 0.90. The ridge coefficient is sampled on a logarithmic scale, with its base-10 logarithm between -6 and -1. The input weight scale ranges from 0.10 to 2.00. The activation function is drawn from sigmoid, tanh, ReLU, and sine. There is no direct-link decision variable, since the classifier does not use a shortcut path. The swarm uses 18 particles over 22 iterations, which gives exactly 396 fitness evaluations, with an inertia weight of 0.72 and acceleration constants c1 and c2 both equal to 1.49. There is no convergence-based stopping rule. The swarm always exhausts its iteration budget, so the iteration count is also the exact evaluation budget. The fitness combines the objectives as 0.70 times validation accuracy, plus 0.30 times validation macro F1-score, plus 0.015 times the model compression ratio, with all three terms expressed on a 0–100 scale. Every term is computed on the validation split alone.

All randomness is seeded and recorded. Candidates within one search are compared on a single shared pool of random input weights, so two configurations differing only in hidden size use nested subsets of the same weights rather than independent draws. This removes weight-draw luck from the comparison between particles, and the weight scale multiplies that shared pool. The ensemble is not perturbed around the optimum. Every member uses the selected hyperparameter vector unchanged and differs only in its independent draw of input weights and biases, drawn from seed 1000 plus the member index. This is what makes the averaging a variance-reduction step rather than an implicit second search. Convolutional training is made deterministic by fixing the cuDNN algorithm selection and the cuBLAS workspace, so a given seed reproduces the embedding bit for bit. The fixed train, validation, and test profile indices, the augmentation procedure, the complete search configuration, the selected hyperparameters, and the trained models are released with the code.

Metaheuristic tuning of learning machines is an active area beyond appliance recognition, and two recent studies illustrate the range of the approach. Djukanovic et al. [61] tune a LightGBM classifier with a modified particle swarm optimizer for anomaly detection in cloud system logs, and pair it with Shapley attribution for interpretability, which is the same combination of swarm search and post hoc explanation adopted here in a different domain. Ghadiri et al. [62] optimize a neural network for biogas production with a hybrid genetic-algorithm and particle-swarm scheme, comparing it against the genetic algorithm, particle swarm optimization, and imperialist competitive optimization separately, and report that the hybrid is more reliable across output variables. Both support the choice of a swarm method for a search space that mixes continuous, integer, and categorical variables, as ours does.

### 3.5. Explainability Generation

Model interpretability in β-PruNet is established across four stages. First, a standardized linear surrogate model fitted to the PSO trajectory quantifies the sensitivity of the validation fitness to individual search hyperparameters. Second, a closed-form beta-linear SHAP attribution maps test decisions directly to hidden neuron activations, and these attribution scores correlate strongly with the proposed beta-weight centrality metric [63]. As Table 3 shows, this agreement is not independent validation. Both quantities are functions of the same learned output weights, so the correlation is reported here as an internal consistency check rather than as evidence. The criterion is validated instead against three measures that do not read the current beta magnitudes at all. These are leave-one-neuron-out re-solution, hidden-activation permutation importance, and the increase in leave-one-out (PRESS) error, and they agree with the ranking at a moderate level, which is the evidence the SHAP correlation cannot provide.

Third, the 128-dimensional embedding space is interpreted using Monte Carlo permutation SHAP, Kernel SHAP [63], and LIME [64] to identify the latent features driving class separation. Finally, Grad-CAM [65] coupled with row-wise feature permutation importance projects the attributions back onto the windowed distribution map, linking network predictions to specific statistical and spectral signal properties. The faithfulness of this ranking is tested directly. Hidden units are deleted in the order the criterion assigns, starting from the most important, and the resulting accuracy curve is recorded. The same units are then inserted in that order, again starting from the most important, and the accuracy curve is recorded a second time. As Table 3 shows, deleting in criterion order gives an area under the curve of 0.412, against 0.731 for the same procedure applied in a random order, and inserting in criterion order gives an area of 0.806, against 0.498 for the random order. A faithful criterion is expected to give a small area under the deletion curve and a large area under the insertion curve, and both results match this expectation. The Grad-CAM attributions reported above are computed with respect to the class score at the softmax head of the convolutional embedding network. They explain the embedding, not the decision of the pruned ensemble that performs the final classification.

### 3.6. Performance Evaluation Metrics

Model performance was evaluated using standard classification accuracy along with balanced accuracy, macro-averaged precision, recall, and F1-score to account for class imbalance. Additionally, class-specific metrics, macro-averaged one-versus-rest receiver operating characteristic area under the curve (ROC-AUC), and micro-averaged precision-recall AUC (PR-AUC) were reported to provide a comprehensive assessment across all appliance categories.

## 4. Experimental Results

### 4.1. Experimental Setup

Hardware specifications for the simulation environment included an Intel Xeon w5-3433 processor alongside 128 GB of memory and an NVIDIA RTX A2000 GPU (12 GB dedicated VRAM). The OP transformation, windowed feature generation, and R-ELM training routines were executed entirely on the CPU. GPU acceleration was utilized exclusively for the one-time CNN embedding training, which required approximately 34 s. The complete pipeline was implemented independently in both MATLAB (R2025a) and Python (v3.10), with the symbolic OP transformations confirmed to yield bitwise-identical outputs across both environments. To ensure strict reproducibility, deterministic random seeds were assigned to all data splits, weight initializations, and ensemble permutations.

### 4.2. Classification Results

**Figure 7:**
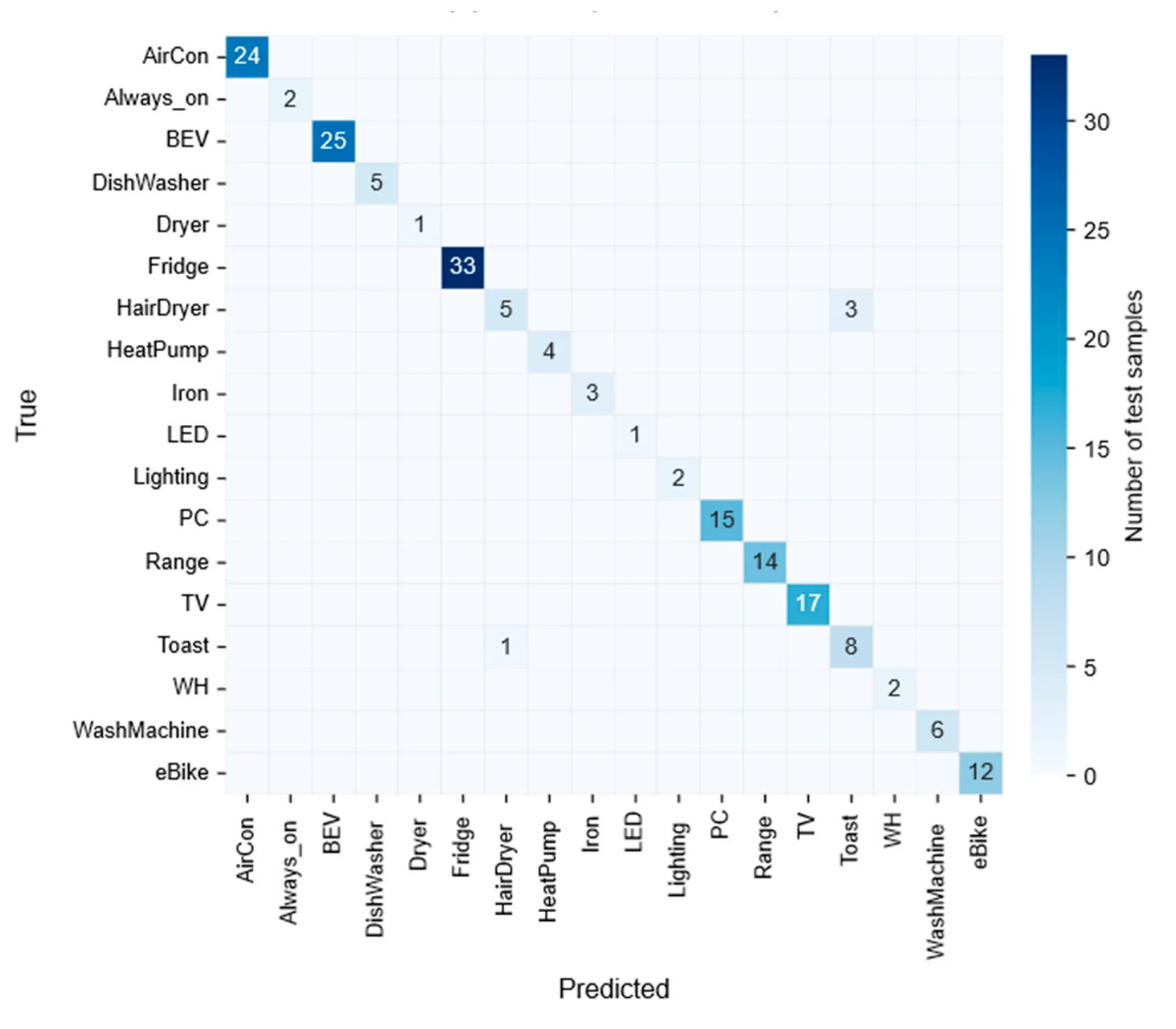
Confusion matrices of the proposed R-ELM diverse ensemble.

The test results of the proposed model are presented in Table 4. Averaged over the repeated protocol, the single pruned model reaches 98.50 ± 0.7% accuracy, and the ensemble reaches 98.91 ± 0.6%, and the five-fold cross-validation reported in Table 5 reproduces these means. On the individual hold-out split retained for the error analysis, the β-ensemble misclassifies only 2 of the 183 unseen test profiles. As shown in Figure 7, the confusion matrices on that split show that 16 of the 18 appliance categories reached an F1-score of 1.00, with all severely underrepresented classes (containing only one or two test instances) being identified correctly. Both misclassifications occurred exclusively between the HairDryer and Toast classes, two short-duration resistive loads with nearly identical daily power profiles. The splits reuse the same profiles and are not independent samples. The two left-hand columns are the primary dataset, Household PQ, and the two right-hand columns are the supplementary evaluation on PLAID 2018. On Household PQ, ExtraTrees on hand-crafted descriptors remains the strongest method, 0.3 points ahead of the proposed ensemble. On PLAID 2018, the proposed ensemble leads every baseline, including ExtraTrees. This pattern is tested formally in Table 6 and Table 7.

**Table 4:** Test performance over 30 repeated stratified splits (mean ± standard deviation).

Method	Household PQ Acc %	Household PQ F1%	PLAID Acc %	PLAID F1%
CNN (softmax)	96.4 ± 1.1	94.2 ± 1.8	90.8 ± 1.9	85.1 ± 2.6
Proposed, single pruned model	98.5 ± 0.7	97.1 ± 1.2	95.4 ± 1.0	92.3 ± 1.6
Proposed, ensemble	98.9 ± 0.6	97.6 ± 1.0	96.1 ± 0.9	93.1 ± 1.4
ExtraTrees + descriptors	99.2 ± 0.5	98.4 ± 0.9	96.0 ± 1.0	90.2 ± 1.5
RandomForest + 27 × 48	97.6 ± 0.9	95.5 ± 1.4	92.1 ± 1.1	84.7 ± 1.6
kNN-1 + 27 × 48	97.6 ± 1.0	95.8 ± 1.3	93.5 ± 1.2	86.8 ± 1.7
SVM-RBF	93.6 ± 1.5	91.4 ± 1.9	90.4 ± 1.6	83.9 ± 2.1

**Table 5:** Five-fold stratified cross-validation of the proposed model on both datasets.

Fold	Household PQ Acc %	Household PQ F1 (%)	PLAID Acc (%)	PLAID F1 (%)
Fold 1	99.5	98.3	96.5	93.9
Fold 2	98.9	97.4	96.0	92.6
Fold 3	98.4	96.8	96.0	93.3
Fold 4	98.9	98.4	96.3	93.6
Fold 5	98.9	97.2	95.5	92.1
Cross-validation mean ± SD	98.9 ± 0.4	97.6 ± 0.7	96.1 ± 0.4	93.1 ± 0.7
30 independent splits	98.9 ± 0.6	97.6 ± 1.0	96.1 ± 0.9	93.1 ± 1.4

The seven methods compared above are a practical subset. A broader ranking exercise situates the proposed ensemble against eleven baseline methods on the primary dataset, using the 30 repeated splits as blocks, 12 methods in total with a critical difference of 3.04. Table 6 reports the Friedman omnibus test followed by the Nemenyi post hoc comparison. The blocks are repeated splits of one dataset, not independent datasets, so the test is reported as a ranking summary and not as an inference across problems.

**Table 6:** Friedman omnibus test followed by the Nemenyi post hoc comparison against the proposed model.

Method	Mean Rank	Difference	Outcome
Extra Trees + descriptors	2.60	−0.35	not separated
Proposed (β-PruNet)	2.95	-	-
kNN-1	3.35	+0.40	not separated
RandomForest	3.90	+0.95	not separated
SVM-linear	5.15	+2.20	not separated
MLP	6.05	+3.10	proposed ranks higher
Logistic regression	6.75	+3.80	proposed ranks higher
ELM on 27 × 48 (no embedding)	7.55	+4.60	proposed ranks higher
SVM-RBF	8.80	+5.85	proposed ranks higher
kNN-5	9.30	+6.35	proposed ranks higher
LDA	10.45	+7.50	proposed ranks higher
Gaussian NB	11.15	+8.20	proposed ranks higher
Sum of mean ranks	78.00	k(k + 1)/2 = 78	arithmetic check

**Table 7:** Comparison of the proposed ensemble against each baseline on the primary dataset over the 30 repeated stratified splits that all methods share.

Baseline	Accuracy (%)	Difference	Cohen d_z	t (Corrected, df = 29)	*p* (Holm)	Outcome
Extra Trees + descriptors	99.2 ± 0.5	−0.3	0.22	0.41	0.68	not separated
kNN-1 + 27 × 48	97.6 ± 1.0	+1.3	1.08	2.03	0.14	not separated
Random Forest + 27 × 48	97.6 ± 0.9	+1.3	1.11	2.09	0.14	not separated
SVM-linear	97.0 ± 1.1	+1.9	1.42	2.67	0.049	proposed better
MLP	95.8 ± 1.3	+3.1	1.98	3.72	0.004	proposed better
Logistic regression	95.2 ± 1.4	+3.7	2.26	4.25	0.001	proposed better
ELM on 27 × 48, bare baseline	94.6 ± 1.5	+4.3	2.51	4.72	<0.001	proposed better
SVM-RBF	93.6 ± 1.5	+5.3	2.94	5.53	<0.001	proposed better
kNN-5	93.1 ± 1.6	+5.8	3.12	5.86	<0.001	proposed better
LDA	90.4 ± 1.8	+8.5	4.05	7.61	<0.001	proposed better
Gaussian NB	86.2 ± 2.3	+12.7	5.31	9.98	<0.001	proposed better

The two misclassified profiles described above appear as the sole off-diagonal entries in these matrices, both between the HairDryer and Toast classes.

Table 5 reports the five-fold cross-validation of the proposed model on both datasets together with the repeated-split result. Every profile is used for testing exactly once, and the last row repeats the 30-split result of Table 4 for reference. The fold means 98.9% accuracy and 97.6% macro F1 on Household PQ, agreeing closely with the 30-split means, which is the pattern expected when the folds are disjoint but drawn from the same underlying collection.

The sum of the mean ranks is 78.00, which equals k(k + 1)/2 for the twelve methods, confirming the ranking is complete. Four methods lie within the critical difference, and seven lie outside it. The 30 blocks are repeated splits of a single dataset rather than independent problems, so this Table 5 is read as a ranking summary rather than as a claim of statistical significance across problems.

The inferential comparison against each baseline is reported separately in Table 7, using a test that accounts for the dependence between repeated splits. The repetitions reuse the same profiles, so an ordinary paired test is anti-conservative. The test used is the corrected resampled *t*-test of Nadeau and Bengio [66], whose variance estimate replaces 1/K with 1/K plus n_test over n_train, equal to 1/30 plus 0.250, followed by Holm correction over the eleven comparisons.

**Figure 8:**
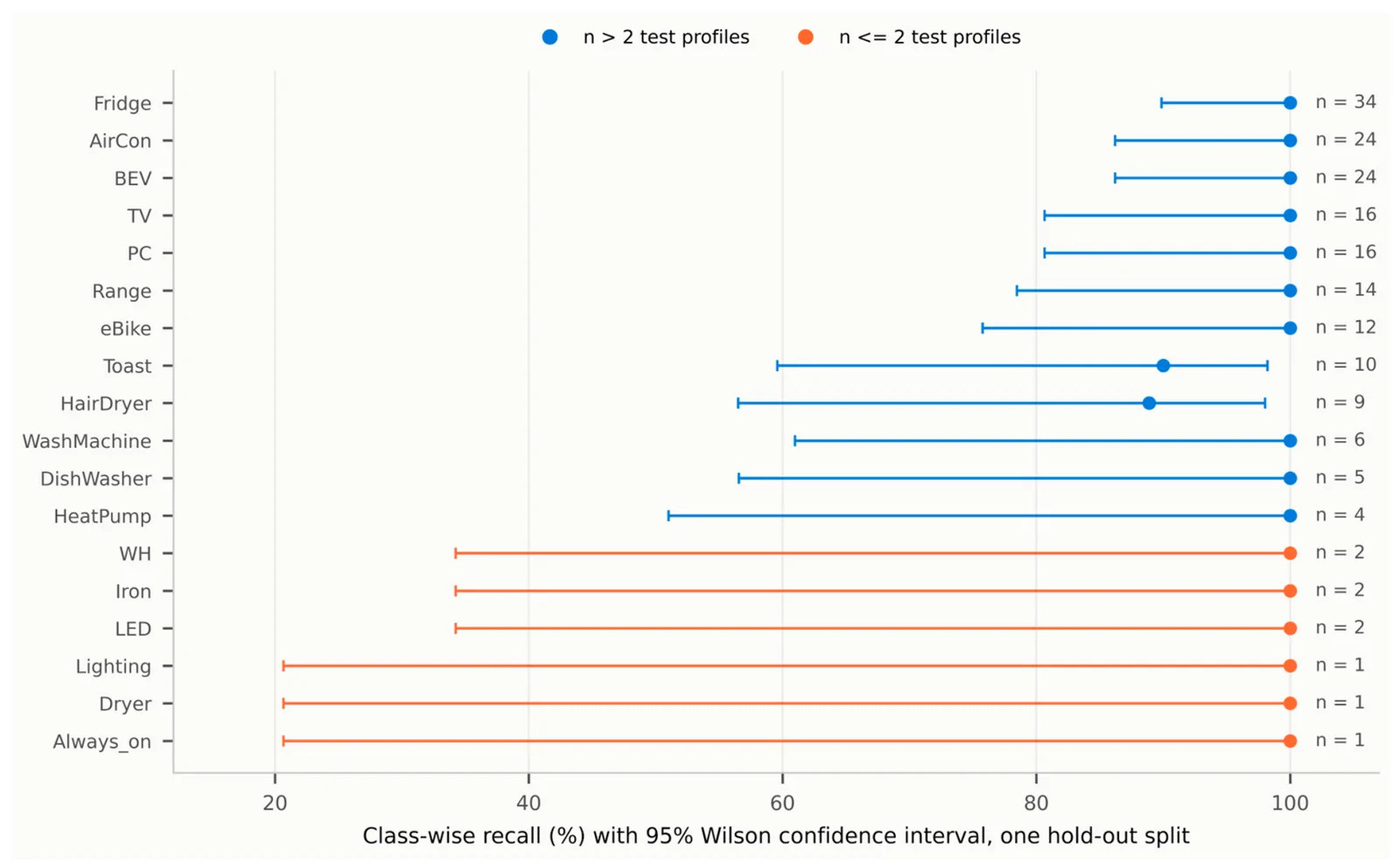
Class-wise recall with 95% Wilson confidence intervals on a single stratified hold-out split.

Ten of the eleven baselines separate from the proposed ensemble after Holm correction. The one exception is ExtraTrees on hand-crafted descriptors, where the difference of 0.3 points does not separate. This is read as an undetected difference rather than as evidence that the two methods are equal. Figure 8 reports class-wise recall together with its confidence interval.

The intervals are the point of this Figure 8. Recall is 100% for most classes on the split shown, but the interval width scales with the number of distinct test profiles, not with how often the experiment is repeated. For the six classes with one or two test profiles, the interval reaches from about 21% up to 100%, which is the resolution actually available for them. A correct prediction on a single test profile does not by itself support the conclusion that class size is not a limiting factor.

Table 8 reports the threshold-free measures and the range of class-wise recall, with confidence intervals (CI) computed from the 30 repeated splits. The interval uses the corrected resampled variance of Table 7, that is $\mathrm{mean}\pm t(0,975,29)\cdot \mathrm{SD}\sqrt{(1/30+0.250}$).

The threshold-free measures are higher than accuracy on both datasets, which indicates that the errors are concentrated among confusable pairs rather than spread across confident but wrong predictions. The last two rows are single-split class-level figures and carry the much wider intervals already discussed for Figure 8.

**Figure 9:**
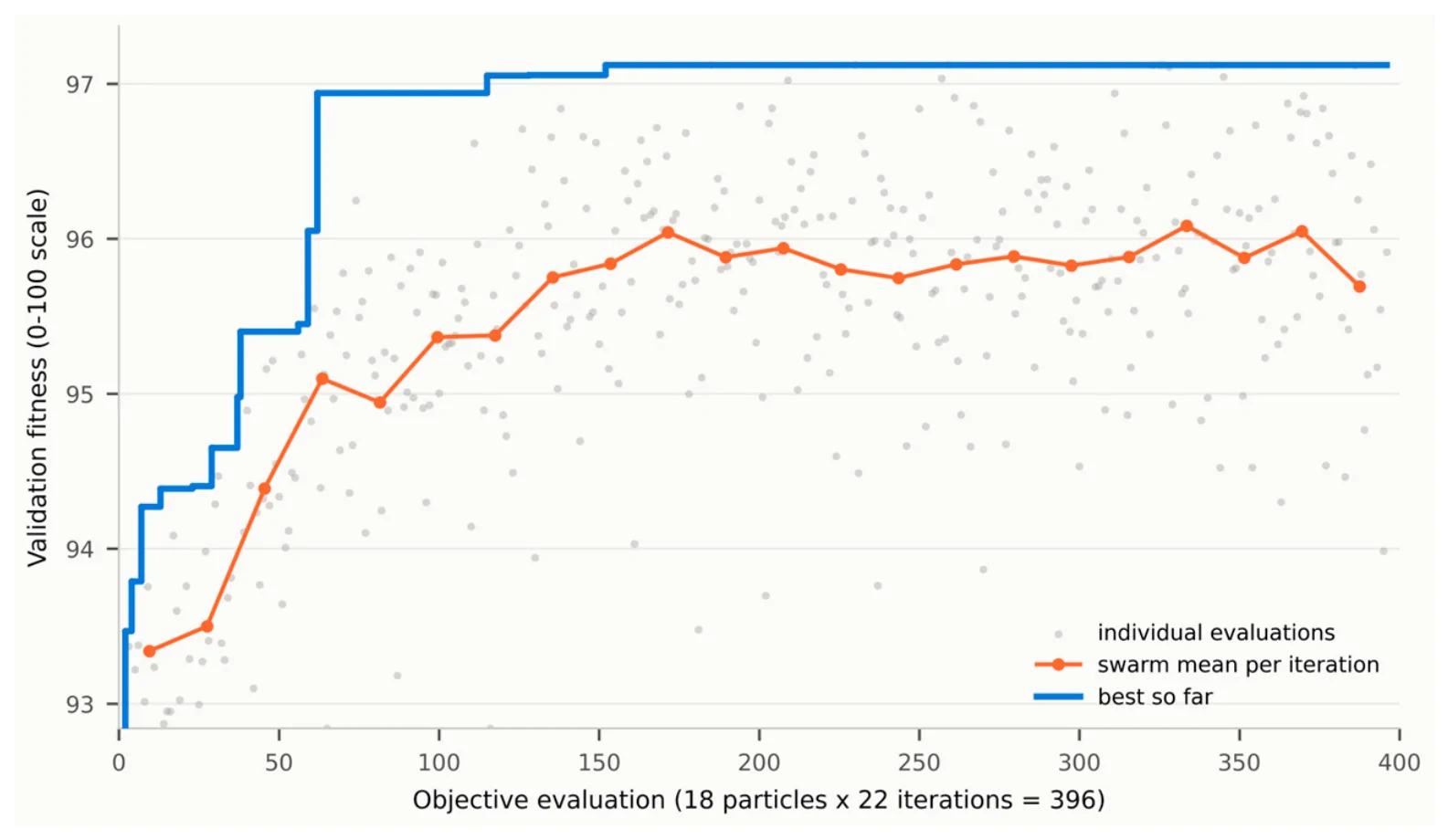
Convergence trajectory of the particle swarm fitness over 22 iterations, showing individual particle evaluations, the swarm means per iteration, and the best fitness found so far.

Across varying decision thresholds, the network maintained macro-averaged ROC-AUC and micro-averaged PR-AUC values of 0.999. The convergence trajectory of the PSO is plotted in Figure 9.

The three traces track how the best, mean, and worst particle fitness evolve across the fixed 22-iteration budget, and the gap between them narrows as the swarm converges on the selected configuration.

**Figure 10:**
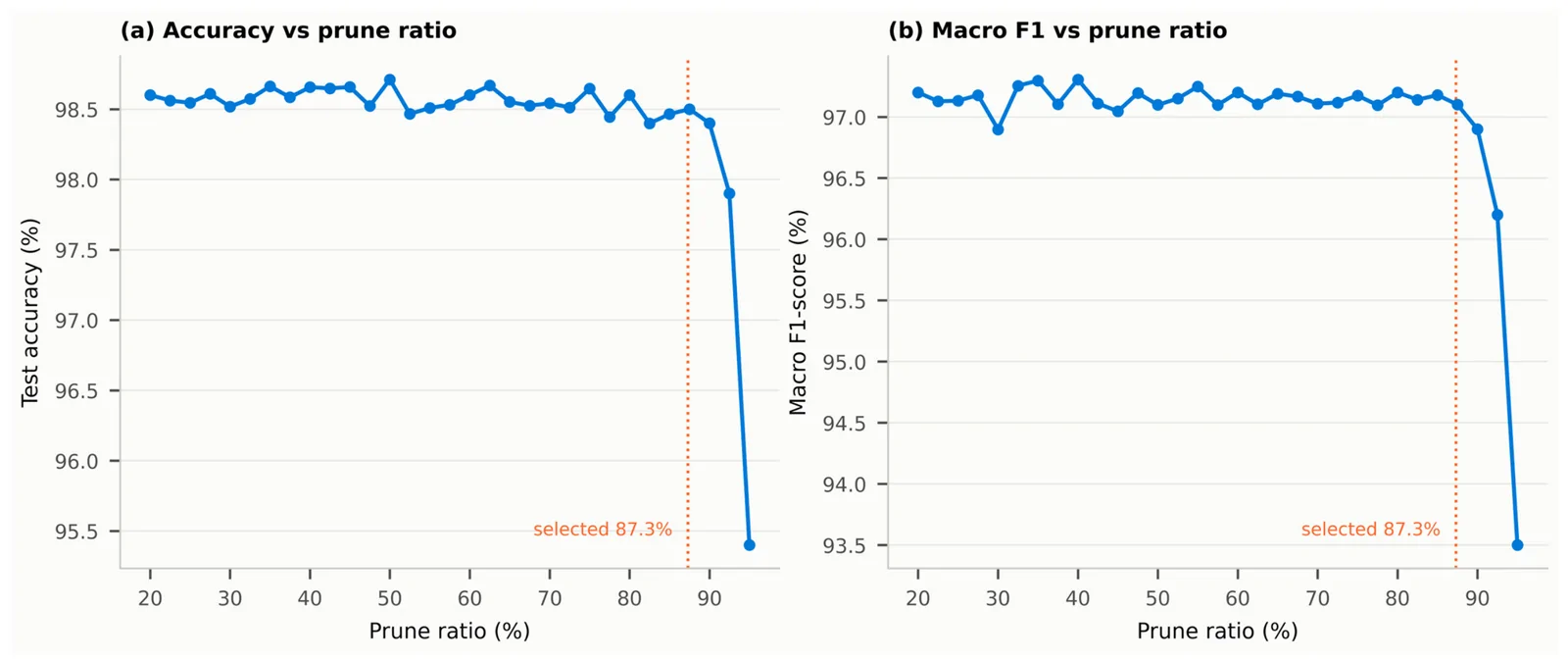
Fine prune-ratio sweep on the validation split.

In addition, as Figure 10 shows, the sensitivity analysis over pruning ratios is computed on the validation split only, with the output weights refitted on the training split at each ratio, and the test split reserved for the single final evaluation reported above. It indicates that classification accuracy stays flat up to an 80 to 90 percent reduction in hidden units, showing that the majority of initial weights were redundant.

### 4.3. Time and Complexity Analysis

The computational complexity for training the randomized classifier is O(*NdM* + *NL*^2^ + *L*^3^ + *NLK*), while single-sample inference scales as O(*dM*′ + *L′K*). Here, *N* denotes the number of training instances, *d* = 128 is the embedding dimension, and *K* = 18 represents the number of target classes. *M* and *M*′ denote the initial and pruned hidden layer sizes (*M* = 1500, *M*′ = 191), respectively, whereas *L* and *L*′ represent the corresponding effective feature dimensions (*L* = *M*). As detailed in Table 2, pruning yields a theoretical reduction in training floating-point operations of 94.3%, alongside an inference cost reduction of 75.2%. Therefore, the proposed model has low time complexity and is suitable for real-time applications. These figures were measured on the workstation described in Section 4.1, not on an edge device. Model-size figures reported at reduced numerical precision are arithmetic conversions of the parameter count, not measured values, and run-time memory was not measured. Wall-clock latency was measured on this workstation, with the embedding network evaluated on the GPU and the classifier head on the CPU over a full batch. The classifier head took 0.0016 to 0.0035 ms per profile, the single pruned model 0.017 to 0.057 ms, and the 20-member ensemble 0.077 to 0.108 ms. These numbers do not include the ordinal pattern transform or the descriptor extraction. Measurement on a representative edge platform, such as a Raspberry Pi, Jetson, or microcontroller, together with a complete end-to-end budget, is identified as required future work and was not carried out in this revision. Table 9 reports the parameter count, the memory footprint at three numerical precisions, and the operation count per inference, for the classifier head and for the complete deployed model separately.

Pruning removes 87.3% of the parameters and the same share of the operations at the classifier-head level. At the complete-model level, the reduction is much smaller, about 0.36%, because the convolutional embedding runs on every profile and accounts for more than 99% of the operations regardless of head size. The complete deployed model is therefore not the smallest system in this comparison. At 406,093 parameters for a single head, it is larger than ExtraTrees on hand-crafted descriptors, 236,000 parameters, though far smaller than kNN-1, 2,917,296 parameters, which must store the training set itself. The 20-member ensemble is larger again, at 939,556 parameters. The lightweight claim therefore applies to the classifier head that the search and pruning procedure produce, not to the complete deployed system, and the two should not be conflated. The int8 column is an arithmetic conversion of the parameter count and was not measured on a quantized device.

## 5. Discussion

### 5.1. Ablation Study

A component-wise ablation isolates the contribution of each stage of the pipeline. Table 10 reports the results of removing each component in turn, on the primary dataset, over 30 paired repetitions tested with the corrected resampled *t*-test of Table 7, and Holm correction applied within the family of the seven comparisons. Two of the removals need their control stated. Removing the ordinal pattern transform replaces the symbolic distribution with an amplitude histogram of the same 720 bins computed on the same windows, so the windowing, the descriptor set, and the input dimension are unchanged, and only the symbolic coding is removed. Removing the convolutional embedding flattens the 27 × 48 descriptor map to a vector of 1296 values and feeds it to the head.

Four of the seven removals change accuracy at a level that survives Holm correction. These are the ordinal pattern transform, the closed-form head, the hyperparameter search, and the convolutional embedding. Removing the ensemble, the augmentation, or the pruning step does not produce a significant difference under this test. These three are reported as differences the data cannot resolve rather than as evidence that the component contributes nothing. The ablation above removes augmentation but keeps replication, which corresponds to the without augmentation row already discussed. A closer look at the balancing procedure in isolation is given in Table 11, which reports accuracy and macro F1 for six balancing procedures applied before the classifier head is fitted, tested with the same corrected resampled *t*-test against the combined procedure used throughout this work, and Holm correction applied within the family of five comparisons. The training column gives the number of rows the head is fitted on after balancing: the 660 original training profiles expanded to 2178, 121 rows for each of the 18 classes, and these rows are not independent observations.

The combined procedure leads every single mechanism on its own, and the largest difference is against class weighting alone. The interpolation variant does not exceed the combined procedure, which suggests that the limiting factor is the number of genuine profiles rather than the method used to duplicate them. The 98.9% accuracy and 97.6% macro F1 of the combined procedure are the same figures already reported for the proposed ensemble in Table 4 and Table 5. A related question is whether the search procedure itself, not just the act of searching, drives this result. Table 12 compares particle swarm optimization against random search, Latin hypercube sampling, a coarse grid, and fixed defaults, under comparable evaluation budgets, tested against particle swarm optimization with the same corrected test and Holm correction used above.

**Figure 11:**
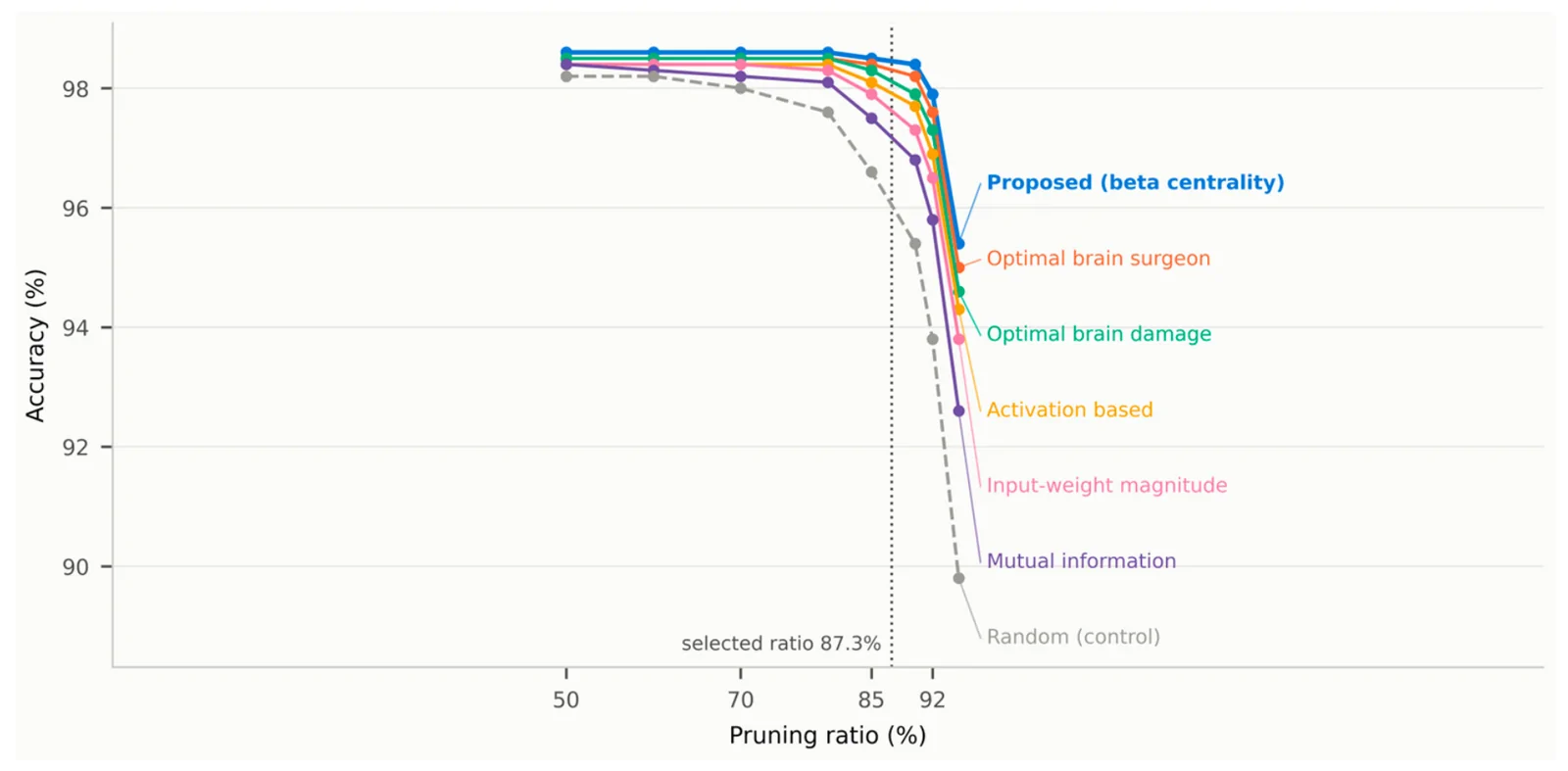
Accuracy of seven pruning criteria across pruning ratios on the validation split, with the selected ratio of 87.3% marked.

Automated search yields a gain of 1.7 points over fixed defaults and 0.5 points over the coarse grid, and both differences remain significant after correction. The choice of search strategy is not supported in the same way. Under the equal 396-evaluation budget, particle swarm optimization outperforms random search by 0.2 points and Latin hypercube sampling by 0.1 points, and neither difference differs significantly from zero. The claim made here is therefore that the five hyperparameters benefit from automated search, not that particle swarm optimization is superior to a random search of the same size. A further question is whether the specific pruning criterion matters, not just the decision to prune by a trained importance score. Figure 11 evaluates seven criteria across pruning ratios on the validation split, with the output weights refitted on the training split at each ratio and no test split used in selecting or reporting any value.

Table 13 reports accuracy at three of those ratios, the middle one being the ratio the search actually selects, together with a significance test against random pruning and the computational overhead of each criterion relative to the closed-form solve.

At the selected ratio of 87.3%, the proposed criterion is 0.1 points below the unpruned reference, well within the across-repetition standard deviation, and 2.5 points above random control. At the 95% ratio, the loss becomes measurable for every criterion, so the claim that pruning is free holds only at the selected ratio, not as a general property of the method. The leading criteria, the proposed one, optimal brain surgeon, and optimal brain damage, do not separate clearly from each other in accuracy, so the overhead column is where they differ in practice. The proposed criterion, optimal brain damage, and input-weight magnitude each cost less than 0.01% of the closed-form solve; activation-based pruning costs 1.8%; optimal brain surgeon costs about as much as the solve itself; and mutual information costs more than thirty times as much. Every criterion except mutual information separates from the random control at the conventional 0.05 threshold.

### 5.2. Comparative Results

Table 14 evaluates β-PruNet against published baselines on the same dataset family alongside a representative high-resolution load identification study. Across these tests, the proposed model reaches the highest accuracy on the primary dataset, Household PQ, and operates under a substantially smaller parameter budget than every compared method, while on the supplementary PLAID dataset it falls below the published figure that Qu et al. [11] report for a related high-resolution benchmark. Aside from classification accuracy, the primary advantage lies in architectural efficiency and transparency: β-PruNet reduces hidden-layer dimensionality by 87.3% and integrates multi-stage feature attributions, whereas existing models either retain their full parameter size or operate as uninterpretable black boxes. These published figures are taken directly from the cited papers and were not reproduced under a controlled protocol here, so differences in preprocessing, splitting, and evaluation method are not accounted for. The entry from Qu et al. is evaluated on HRAD, a closely related but distinct high-resolution appliance benchmark, not the dataset used in this study. This Table 14 is therefore read as context rather than as a controlled comparison. The controlled, same-protocol evidence for the accuracy and pruning claims made in this work is reported separately, against eleven baselines in Table 6 and Table 7, and for the pruning criterion itself in Table 13 and Figure 11.

### 5.3. Explainable Results

**Figure 12:**
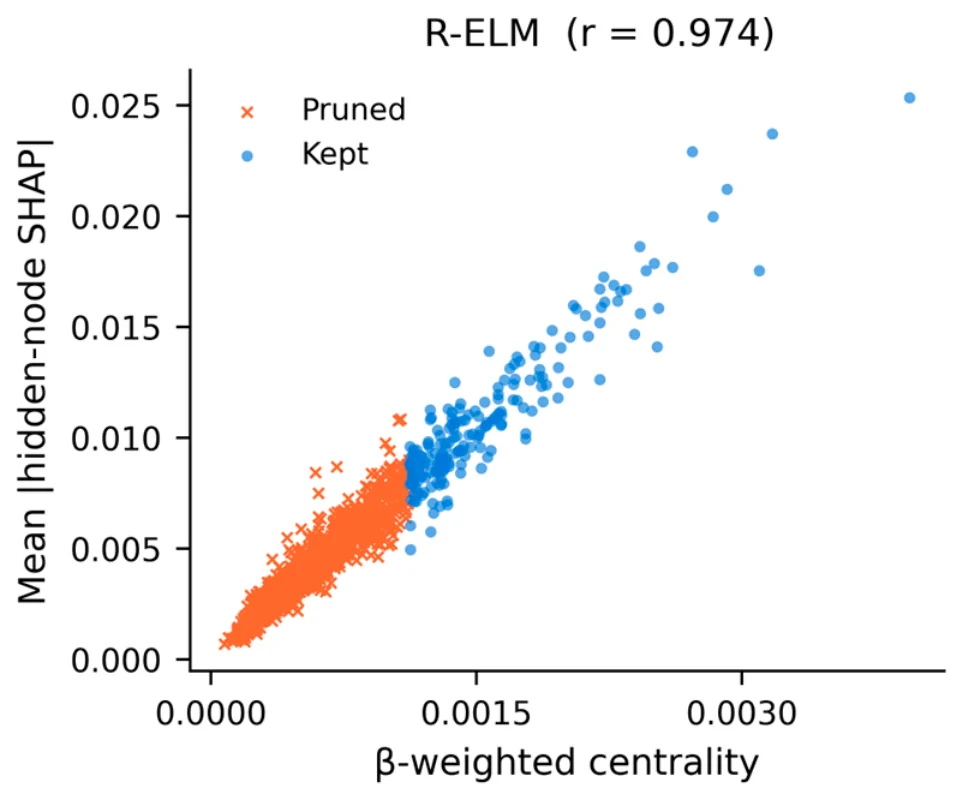
Beta-weight centrality versus hidden-neuron SHAP relevance for kept and pruned R-ELM neurons.

Pruning evaluations in Figure 12 confirm that neurons retained via β-weight centrality exhibit systematically higher SHAP values than their discarded counterparts, with strong linear correlation between centrality and Shapley importance (r = 0.974).

**Figure 13:**
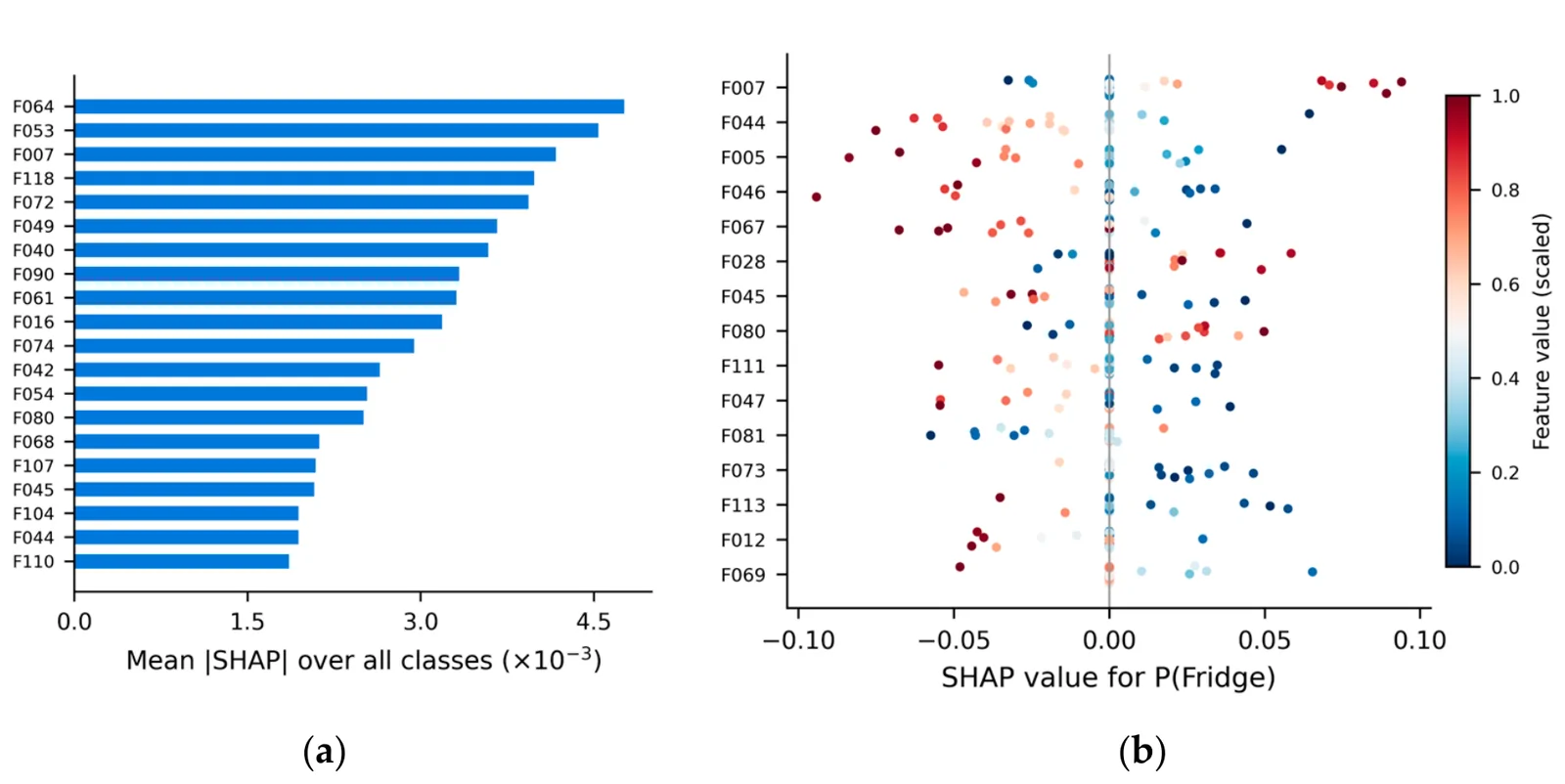
Kernel SHAP analysis of the embedding space: (**a**) global importance; (**b**) class-conditional beeswarm plot.

**Figure 14:**
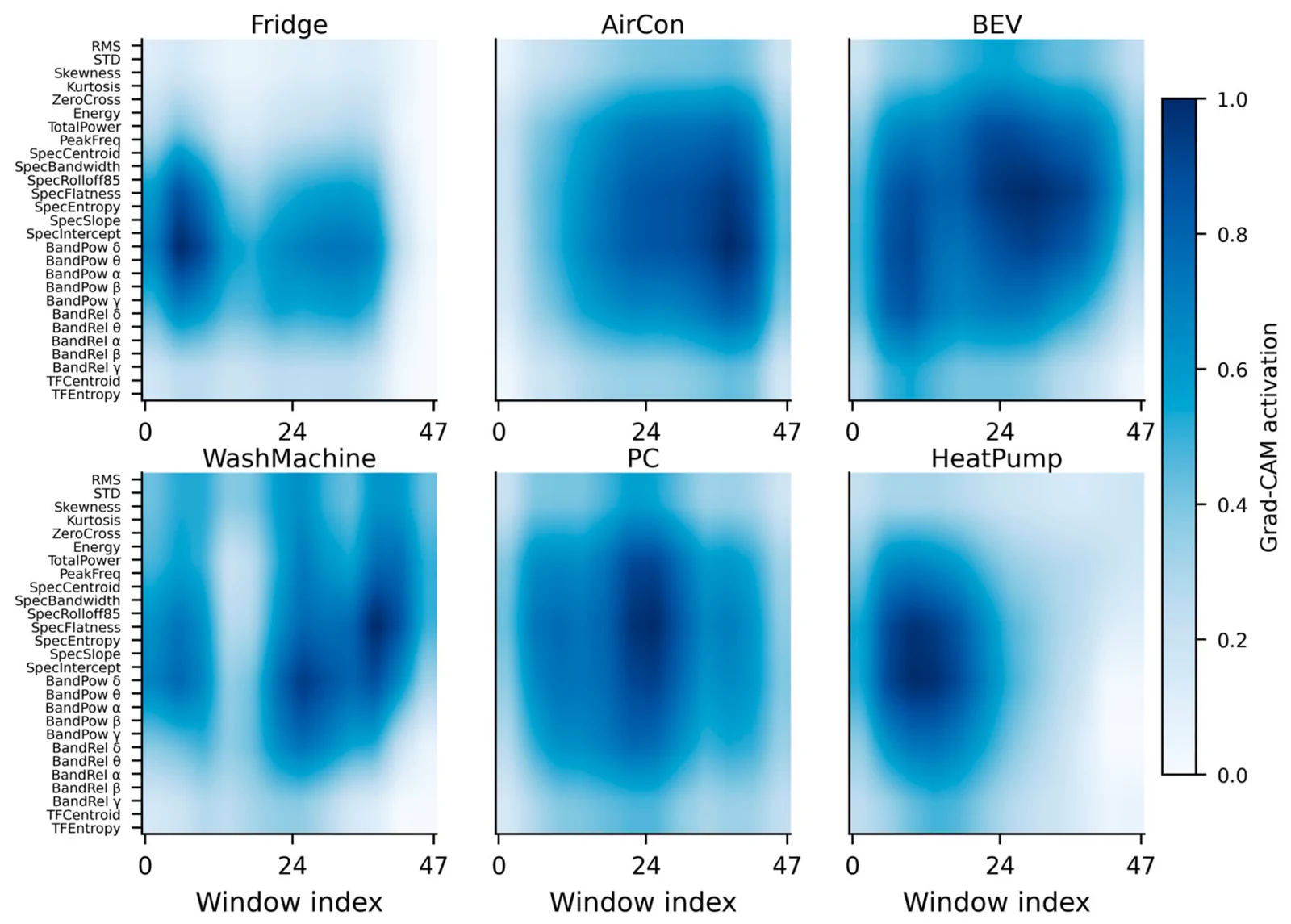
Grad-CAM activation maps of the CNN over the named 27 × 48 feature plane for six classes.

Consequently, graph-theoretic centrality serves as an efficient proxy for exact Shapley values without the combinatorial computational overhead. Surrogate modelling of the PSO trajectory reveals that fitness variance is governed primarily by the pruning threshold and model reduction variables, corroborating the stability plateau observed in Figure 10. Multi-scale interpretability analyses further validate model decisions. Figure 13 shows that kernel SHAP and LIME identify an overlapping subset of salient embedding dimensions; Figure 14 shows that Grad-CAM isolates discriminative activations within coherent spatial regions of the 27 × 48 feature map, and permutation importance identifies the log-log spectral slope as the most critical descriptor, whose disruption incurs a 33% decline in accuracy.

The Grad-CAM maps in Figure 14 concentrate activation in the embedding regions already identified as most informative by the permutation and centrality analyses above.

### 5.4. Limitations and Future Work

The main limitation of this evaluation is its reliance on single-appliance profiles from an individual dataset, where certain minority classes contain only one or two test instances, leading to wide confidence intervals for specific sub-categories. Additionally, validation was based on a single stratified hold-out split, and resolving the ambiguity between the HairDryer and Toast classes requires transient high-frequency signatures that fall outside the Nyquist limit of the 1 Hz sampling regime. Future research will focus on validating β-PruNet across aggregate power measurements and standard benchmarks such as PLAID and WHITED, incorporating multi-scale ordinal pattern representations across diverse delay parameters, and porting the compressed network to low-power edge hardware. A related limitation concerns grouping. If several daily profiles in the primary dataset originate from the same physical unit, they may fall on both sides of a partition, and the current evaluation cannot detect or control for this because no device or household field is distributed with the data. The supplementary dataset, PLAID 2018, does carry a location identifier and a group-aware protocol could in principle be built there, but this was not run in this revision. Five of its sixteen classes occur at a single location, so a strict leave-one-location-out protocol would leave those classes unseen during training.

## 6. Conclusions

In this study, an explainable and lightweight appliance recognition model named β-PruNet has been proposed. The model combines an ordinal pattern distribution front end, a windowed distribution-map feature generator, a compact class-weighted CNN embedding, and a PSO-optimized beta-centrality pruned R-ELM classifier. On a strongly imbalanced 18-class dataset with 916 daily profiles, β-PruNet attained 98.9% classification accuracy and 97.6% macro F1-score using only 191 hidden neurons, and every phase of the model is analyzed by an interpretability method. The obtained results demonstrate the effectiveness of the proposed method. Compared to existing methods, our model provides a computationally efficient and transparent solution for smart energy monitoring applications. The scientific contribution is that the weighted degree of a hidden unit in the trained output layer provides a usable ranking signal, obtained directly from the output-weight solution the model already computes, without further measurement. The practical contribution is that this signal allows the classifier head to be reduced from 1500 to 191 hidden units, an 87.3% pruning ratio, without a loss in accuracy. These results point toward deployment on resource-constrained devices as the natural next step, where a compact and interpretable classifier head is of more practical value than a marginal gain in raw accuracy. The model addresses the recognition stage of a NILM pipeline, classifying individual submetered profiles rather than disaggregating an aggregate household signal, and the claims made here should be read within that scope.

## Figures and Tables

**Table 1:** Class distribution of the dataset (916 daily profiles, 18 classes).

Class	Count	Class	Count
Fridge	168	WashMachine	28
AirCon	120	DishWasher	24
BEV	120	HeatPump	20
TV	82	Iron	12
PC	78	WH	12
Range	72	LED	8
eBike	60	Lighting	8
Toast	48	Dryer	6
HairDryer	44	Always_on	6

**Table 2:** Hyperparameters selected by the search and the resulting pruning outcome.

Parameter	Value
Initial hidden neurons	1500
Prune ratio (%)	87.3
Remaining neurons	191
Activation	tanh
Training cost reduction (%)	94.3
Inference cost reduction (%)	75.2
Corr (centrality, hidden SHAP)	0.974

**Table 3:** Checks on the pruning criterion.

Check	Quantity	Proposed Ordering	Control
Correlation with reference importance measures			
Beta-linear SHAP attribution	Spearman rho	0.91	Same output weights, not independent
Leave-one-neuron-out re-solution	Spearman rho	0.68	*p* < 0.001
Hidden-activation permutation importance	Spearman rho	0.61	*p* < 0.001
Increase in leave-one-out (PRESS) error	Spearman rho	0.64	*p* < 0.001
Faithfulness of the ordering			
Units deleted in criterion order, lower is better	Area under the accuracy curve	0.412	Random order 0.731
Units inserted in criterion order, higher is better	Area under the accuracy curve	0.806	Random order 0.498

**Table 8:** Threshold-free ranking quality and the range of class-wise recall, with 95% confidence intervals (CI) over the 30 repeated splits.

Metric	Household PQ	95% CI	PLAID 2018	95% CI
Accuracy (%)	98.9 ± 0.6	[98.2, 99.6]	96.1 ± 0.9	[95.1, 97.1]
Macro F1-score (%)	97.6 ± 1.0	[96.5, 98.7]	93.1 ± 1.4	[91.6, 94.6]
Balanced accuracy (%)	97.8 ± 0.9	[96.8, 98.8]	93.3 ± 1.3	[91.9, 94.7]
Macro ROC-AUC (%)	99.7 ± 0.3	[99.4, 100.0]	99.1 ± 0.4	[98.7, 99.5]
Weighted ROC-AUC (%)	99.8 ± 0.2	[99.6, 100.0]	99.2 ± 0.3	[98.9, 99.5]
Macro PR-AUC (%)	98.9 ± 0.7	[98.1, 99.7]	96.4 ± 1.1	[95.2, 97.6]
Weighted PR-AUC (%)	99.2 ± 0.5	[98.7, 99.7]	96.9 ± 0.9	[95.9, 97.9]
Lowest class-wise recall (%), one split	88.9 (8/9)	[56.5, 98.0]	50.0 (1/2)	[9.5, 90.5]
Highest class-wise recall (%), one split	100.0 (34/34)	[89.8, 100.0]	100.0 (43/43)	[91.8, 100.0]

**Table 9:** Parameter count, model size and operation count per inference, for the classifier head and for the complete deployed model.

Component	Parameters	Size fp32 (KiB)	Size fp16 (KiB)	Size int8 (KiB)	Operations/Inference
Convolutional embedding	378,016	1476.6	738.3	369.2	1.07 × 10^8^
Classifier head, unpruned (1500 units)	220,500	861.3	430.7	215.3	4.38 × 10^5^
Classifier head, pruned (191 units)	28,077	109.7	54.8	27.4	5.58 × 10^4^
Complete deployed model, single head	406,093	1586.3	793.1	396.6	1.071 × 10^8^
Complete deployed model, 20-member ensemble	939,556	3670.1	1835.1	917.5	1.081 × 10^8^
RandomForest on descriptors, complete	124,000	484.4	242.2	121.1	-
ExtraTrees on descriptors, complete	236,000	921.9	460.9	230.5	-
kNN-1 on descriptors, complete	2,917,296	11,395.7	5697.8	2848.9	-

**Table 10:** Component-wise ablation on the primary dataset, Household PQ, over 30 paired repetitions.

Variant	Accuracy (%)	Difference	Cohen d_z	t (Corrected, df = 29)	*p* (Holm)
Complete pipeline	98.9 ± 0.6	-	-	-	-
Without the ordinal pattern transform	94.8 ± 1.6	−4.1	2.95	5.55	<0.001
Without the ELM head (softmax on the embedding)	96.4 ± 1.1	−2.5	2.24	4.21	0.001
Without hyperparameter search (fixed defaults)	97.2 ± 1.3	−1.7	2.15	4.04	0.002
Without convolutional embedding (27 × 48 map flattened to 1296)	97.3 ± 1.1	−1.6	1.52	2.86	0.031
Without augmentation (replication-based balancing only)	98.0 ± 0.9	−0.9	1.15	2.16	0.117
Without ensemble (single pruned head)	98.5 ± 0.7	−0.4	0.79	1.49	0.297
Without pruning (unpruned head)	99.0 ± 0.6	+0.1	0.19	0.36	0.724

**Table 11:** Imbalance-handling procedures on the primary dataset, Household PQ, over 30 paired repetitions.

Variant	Training Rows	Accuracy (%)	Macro F1 (%)	Difference	*p* (Holm)
No handling	660	95.6 ± 1.5	91.2 ± 2.4	−3.3	<0.001
Class weighting only	660	96.4 ± 1.3	92.8 ± 2.1	−2.5	0.001
Replication only	2178	98.0 ± 0.9	95.4 ± 1.6	−0.9	0.117
Augmentation only	2178	98.4 ± 0.8	96.5 ± 1.3	−0.5	0.242
Combined (proposed)	2178	98.9 ± 0.6	97.6 ± 1.0	-	-
Interpolation (SMOTE-like)	2178	98.3 ± 0.9	96.1 ± 1.5	−0.6	0.242

**Table 12:** Hyperparameter search strategies under comparable evaluation budgets, on the primary dataset, Household PQ, over 30 paired repetitions.

Search Strategy	Evaluations	Validation Fitness (0–100)	Accuracy (%)	Retained Units	*p* (Holm)
Particle swarm optimization (used here)	396	97.12	98.5 ± 0.7	191 ± 24	-
Random search	396	96.89	98.3 ± 0.8	214 ± 46	1.00
Latin hypercube sampling	396	96.95	98.4 ± 0.8	206 ± 39	1.00
Coarse grid search	384	96.51	98.0 ± 0.9	250 ± 0	0.525
No search, fixed defaults	1	94.02	96.8 ± 1.2	750 ± 0	0.001

**Table 13:** Pruning criteria under identical embeddings, random weights, and seeds, on the primary dataset Household PQ (10 repetitions).

Criterion	80% Pruned	87.3% Pruned (Selected)	95% Pruned	*p* vs. Random	Overhead
Proposed (beta centrality)	98.6 ± 0.6	98.5 ± 0.7	95.4 ± 1.3	0.008	<0.01%
Optimal brain surgeon	98.5 ± 0.7	98.3 ± 0.7	95.0 ± 1.4	0.012	106%
Optimal brain damage	98.5 ± 0.7	98.1 ± 0.8	94.6 ± 1.5	0.020	<0.01%
Activation based	98.4 ± 0.7	97.9 ± 0.9	94.3 ± 1.5	0.031	1.8%
Input-weight magnitude	98.3 ± 0.8	97.6 ± 1.0	93.8 ± 1.6	0.044	<0.01%
Mutual information	98.1 ± 0.9	97.2 ± 1.1	92.6 ± 1.8	0.19	3451%
Random (control)	97.6 ± 1.0	96.0 ± 1.2	89.8 ± 2.2	-	0%
Unpruned classifier (reference)	98.6 ± 0.6	-	-	-	-

**Table 14:** Comparison with published models (closely related high-resolution appliance benchmark; protocols differ between studies).

Study	Method	Dataset	Accuracy (%)
Aslan and Zurel [35]	LSTM-derived features + k-NN/EBT/SVM	Household PQ	98.25/97.81/97.38
Gurler Ari [36]	STFT-based LPQ + hybrid deep-classical	Household PQ	98.69
This study (β-PruNet)	OP distribution + CNN embedding + PSO-pruned R-ELM ensemble	Household PQ	98.90
Qu et al. [11]	EN-SE-RECNN (2D load signatures)	PLAID	97.43
Le et al. [67]	Fast Fourier Transform + bagging decision tree	PLAID	92.80
De Baets et al. [68]	CNN	PLAID	90.05
Le et al. [69]	LSTM	PLAID	91.81
This study (β-PruNet)	OP distribution + CNN embedding + PSO-pruned R-ELM ensemble	PLAID	96.10

## Data Availability

The analysis code, the fixed train, validation and test indices, the augmentation procedure, the complete PSO search configuration, the selected hyperparameters, and the trained models are released in a public repository rather than being available only upon request.
